# Numerical experimental and theoretical investigation of reinforced concrete elements with rectangular spiral rebar for multi-behavior analysis

**DOI:** 10.1038/s41598-025-29097-w

**Published:** 2025-11-22

**Authors:** Mohammad Shamsi, Mohammad Zakerinejad

**Affiliations:** 1 Department of Civil Engineering, University of Hormozgan, P.O. Box 3995, Bandar Abbas, Iran; 2 Goharzamin Mining and Industrial Company, P.O. Box 571-78185, Sirjan, Iran

**Keywords:** Reinforced concrete, Rectangular spiral rebar, Behavior, Numerical, Experimental, Theoretical, Engineering, Civil engineering, Mechanical engineering

## Abstract

Rectangular Spiral Rebar (RSR) is an innovative alternative to conventional ties, offering simplified construction and enhanced performance in reinforced concrete (RC) members. Despite its advantages, a comprehensive evaluation of RSR under multi-behavioral loading conditions (compression, shear, and seismic) remains unexplored. This study integrates numerical, experimental, and theoretical approaches. Numerical models were developed using Abaqus, SAP2000, and VecTor2 to assess RSR performance under static (compression/shear), lateral quasi-static, and seismic loads (five intensity levels). Experimentally, 14 RC short columns with RSR were tested under monotonic compression, varying cross-sectional geometries (rectangular/square) and reinforcement configurations. The Mander confined concrete model was extended to incorporate RSR, refining predictions for longitudinal rebar, cover concrete, and core concrete behavior. RSR increased compressive strength by 8.4% compared to conventional ties, with more uniform stress distribution. Nonlinear finite element analysis (VecTor2) accurately predicted crack patterns, though computational models and standards (e.g., SMCFT, MPLANE, CEB-FIP, CSA, AASHTO) overestimated shear capacity. RSR confinement produced blunter crack patterns. RSR-confined frames exhibited 8% lower drifts under high-intensity ground motions (PGA = 0.52 g) and required 12% less transverse reinforcement. The proposed theoretical method showed high accuracy (average experimental-to-theoretical capacity ratio: 1.03 for RSR specimens). This study bridges critical gaps in RSR research, demonstrating its multi-behavior efficacy in improving strength, constructability, and seismic resilience. The validated analytical model provide a foundation for code adoption and practical applications.

**Supplementary Information:**

The online version contains supplementary material available at 10.1038/s41598-025-29097-w.

## Introduction

Reinforced concrete (RC) elements exhibit enhanced load-bearing capacity under axial compression when effectively confined laterally. It is well-established that lateral confining stress, generated passively by transverse reinforcement, significantly improves this capacity. Passive confinement through transverse reinforcement—using hoops, spirals, or steel ties—remains the standard approach due to its simplicity and effectiveness. While lateral pressure can be applied through external forces^1–4^, this method is generally impractical for most applications. More commonly, confinement is achieved passively using steel tubes, conventional ties, or more recently developed Fiber Reinforced Polymer (FRP) sheets, which capitalize on concrete’s dilatational behavior under high compression loads^5^. Beyond these conventional methods, advancements incorporating innovative reinforcements^6^, recycled materials^7,8^, alternative additives^9,10^, and industrial by-products^11^ have shown promising results in enhancing the strength and durability of confined concrete. Despite significant innovations in confinement techniques, traditional steel ties continue to dominate as transverse reinforcement for rectangular sections, while hoops and spirals are preferred for circular sections due to their superior performance. The extensive research on confined concrete behavior has produced numerous analytical models for its evaluation. For comprehensive reviews of this research, see references^12–17^.

Continuous Transverse Reinforcement (CTR) systems, particularly Rectangular Spiral Rebar (RSR), have emerged as innovative solutions that enhance both structural performance and constructability of RC members. While circular spirals have long been recognized for providing superior strength and ductility in RC members^18–21^ (a fact acknowledged by design codes like American Concrete Institute (ACI-CODE-318-19R22^22^ through increased performance factors) their rectangular counterparts offer unique advantages for practical applications.

The development of CTR in polygonal and rectangular shapes represents a significant advancement aimed at simplifying RC construction. Pioneering work by Karayannis and Chalioris^23^ demonstrated that RSR could enhance shear strength in RC beams by up to 20% compared to traditional stirrups. These findings were corroborated by Shatarat et al.^24,25^, who confirmed RSR’s effectiveness in improving shear behavior, and by De Corte and Boel^26^, who validated that existing shear design formulas remain applicable to CTR-reinforced beams. Beyond shear performance, research has revealed RSR’s advantages in various loading conditions:

1. Envelope-based lateral quasi-static and Seismic Performance: Kakaletsis and Karayannis^27^ found that RC frames with RSR exhibited comparable behavior to conventional systems under lateral loading. Azimi et al.^28–30^ Karayannis and Sirkelis^31^ demonstrated superior performance in beam-column connections, with enhanced ductility, strength, and energy absorption capacity. Eom et al.^32^ showed that CTR-confined column-foundation systems achieved comparable lateral quasi-static performance while using less reinforcement.
2. Torsional Resistance: Chalioris and Karayannis^33^ revealed that RSR significantly impacts torsional strength, with performance dependent on spiral direction and loading conditions.
3. Constructability Benefits: Practical advantages have been equally compelling. Kang et al.^34^ highlighted how CTR systems reduce reinforcement consumption and construction time, while Shahrooz et al.^35^ documented improved constructability through reduced congestion. These benefits extend to various structural elements: beam-column joints referring to reinforcement details within the joint core (Saha and Meesaraganda^36^, shear walls (Fan et al.^37^, and slabs under impact loads (Al-Dala’ien et al.^38^.

Theoretical advancements have paralleled experimental findings. Zakerinejad and Soltani^39^ developed a Local Stress Field Approach (LSFA) for predicting shear capacity in RSR-reinforced members, while Karayannis^40^ provided mechanistic insights into RSR’s superior joint performance. Numerical studies by Xu^41^ and Chen and Xue^42^ have further expanded understanding of RSR behavior in complex loading scenarios and non-conventional applications like concrete-filled steel tubes. Key observations from these collective studies include:

- RSR provides more uniform confinement compared to discrete ties.
- The continuous nature of RSR improves constructability and reduces placement errors.
- Performance benefits extend across multiple loading conditions (shear, torsion, lateral quasi-static).
- Existing design methods often remain applicable, though some modifications may improve accuracy.

This comprehensive body of research establishes RSR as a viable alternative to conventional reinforcement, offering both performance enhancements and practical construction advantages. However, as will be discussed in the following section, several aspects of RSR behavior require further investigation to fully realize its potential in structural applications.

Despite prior studies on similar configurations, such as rectilinear or rectangular spirals, existing research has certainly not covered all aspects related to the impact of the RSR system on RC elements and structures. There are still many gaps and unanswered questions in this area. Some of the most significant deficiencies include:

- Lack of investigation into the multi-behavior of RC members with RSR confinement under various loading conditions such as shear, compression, envelope-based lateral quasi-static, and seismic loads.
- Experimental examination of RC elements with RSR under compression, considering the effects of cross-sectional geometry, bar diameters and spacing of transverse reinforcement.
- Providing an analytical model to predict the complete axial stress-strain compressive behavior (including peak strength, ductility, and post-peak response) of confined RC with RSR, aiming to enhance existing models and validate them with experimental data.

Building upon the identified research gaps, this study presents a comprehensive investigation of RSR systems with the following key objectives:

1. Experimental evaluation: To systematically examine the compressive behavior of RSR-confined RC short columns under monotonic loading, with direct comparison to conventional tied specimens. The experimental program encompasses:

- Two cross-sectional geometries (square and rectangular).
- Varied transverse reinforcement configurations (spacing and bar diameter).
- Comprehensive instrumentation to capture failure mechanisms.

2. Numerical modeling: To develop and validate advanced finite element models using multiple platforms (Abaqus, VecTor2, and SAP2000) for predicting:

- Axial compression behavior.
- Shear capacity and crack patterns: Comparison of the shear capacity of RC members using different code methods and models proposed by other researchers such as Modified Compression Field Theory (MCFT), Simplified MCFT (SMCFT^43^, the MPLANE^44^ program (a computational tool for RC panel analysis), the VecTor2^45^ software (a nonlinear finite element analysis package for RC structures), the Comité Euro-International du Béton (CEB-FIP^46^, Canadian Standards Association (CSA^47^, American Association of State Highway and Transportation Officials (AASHTO [48]), and examining the cracking pattern of these members under shear.
- Seismic performance across five intensity levels (operational to very strong earthquakes).

The combination of Abaqus (for static compression/shear), VecTor2 (for shear cracking accuracy based on the MCFT and Disturbed Stress Field Model (DSFM)), MPLANE (for shear behavior of of RC panels based on the LSFA), and SAP2000 (for quasi-static/seismic analysis) was selected to leverage their respective strengths in addressing the multi-behavior objectives of this study.

3. Theoretical development: To extend and refine existing confined concrete models, particularly Mander’s approach, by:

- Incorporating RSR-specific confinement effects.
- Developing accurate stress-strain relationships.
- Validating predictions against experimental results.

This study employs a multi-methodological approach combining experimental testing (14 RC column specimens), finite element analysis, analytical model development, and seismic performance evaluation to translate findings into practical recommendations for performance-based design approaches, potential code implementation, and construction optimization. Through this comprehensive investigation, the research aims to provide fundamental understanding of RSR multi-behavior under different loading conditions and present practical guidelines for engineers and code committees. This systematic work addresses critical gaps in RSR research while offering both scientific insights and practical applications for modern RC design.

## Methodology

This study employs a multi-phase approach involving experimental testing, numerical simulation, and analytical modeling to evaluate the performance of RC elements confined with RSR. To provide a clear and structured presentation, the subsequent sections are organized by loading type. Each major section begins with a brief summary of the methodology relevant to that loading scenario—namely, axial compression, static shear, envelope-based lateral static loading, and dynamic seismic analysis—followed by a presentation and discussion of the corresponding results (Table 1). This format is intended to ensure clarity and avoid repetitive details, while maintaining a coherent link between the methodological setup and observed outcomes in each behavioral domain.

**Table 1:** Mapping of methodology and result sections with corresponding introductory statements.

Methodology section (Sect. 2)	Corresponding results section (Sect. 3)
2.1. Preliminary prediction of confinement with RSR based on a simple numerical model	3.1. Preliminary numerical results
2.1.1. static modeling (monotonic compression loading)	3.1.1. static behavior of RC elements with RSR under compression loading
2.1.2. static modeling (monotonic shear loading)	3.1.2. static behavior of RC elements with RSR under shear loading
2.1.3. Quasi-static lateral loading	3.1.3. Quasi-static behavior of RC elements with RSR under lateral loading
2.1.4. dynamic modeling (seismic loading)	3.1.4. dynamic behavior of RC elements with RSR under seismic loading
2.2. Testing program	3.2. Test results
2.3. Mander et al. [49] model and proposed analytical model	3.3. Analytical results

### Preliminary prediction of confinement with RSR based on a simple numerical model

Before conducting any experiments in this research, an initial hypothesis based on several simple numerical nonlinear models was considered to assess the potential impact of RSR under axial and lateral static loads. As previously mentioned, the most likely reason for the improved performance of RC members with CTR under various stresses, compared to members with conventional transverse ties, is the three-dimensional behavior of this type of reinforcement and the change in confinement conditions it induces. Therefore, answering the question of “how CTR alters confinement conditions?” can provide insights into many issues related to RSR systems.

#### Static modeling (monotonic compression loading)

**Fig. 1:**
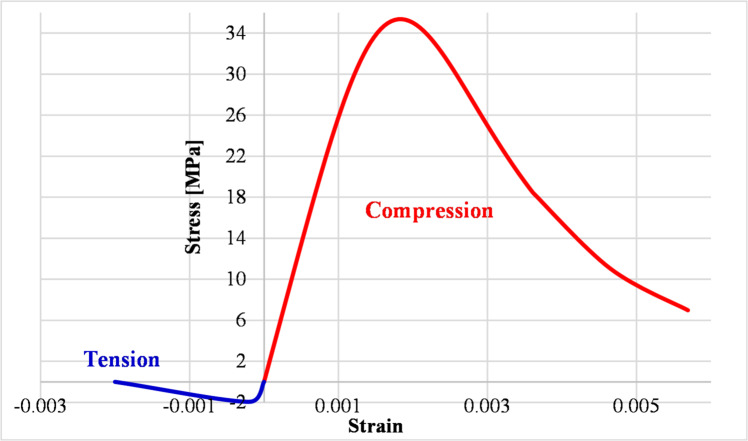
Concrete material behavior.

Finite element modeling using the Abaqus software has been employed to assess the difference in stress distribution between conventional transverse reinforcement and RSR. Two similar concrete core models with a square cross-Sect. (40 cm per side) and a height of 3 m were simulated. The first model used conventional hoops with a cross-sectional area of 1 cm^2^ and a center-to-center spacing of 20 cm, while the second model employed similar continuous transverse reinforcement. To capture post-cracking and inelastic confinement effects, a nonlinear Concrete Damaged Plasticity model was employed for concrete, incorporating both compressive and tensile damage evolution, as show in Fig. 1; Table 2.

**Table 2:** Parameters for concrete damaged plasticity model.

Parameter	Symbol	Value
Dilation angle (˚)	ψ	36
Eccentricity	ε	0.1
Biaxial-to-uniaxial compressive strength ratio	σ_bo_/σ_co_	1.16
Failure surface in the deviator plane normal to the hydrostatic axis	K_c_	0.67
Viscocity parameter	µ	0

The constitutive parameters were extracted from the validated Abaqus simulations of [50,51]. The steel reinforcement was modeled as bilinear elastic–plastic with isotropic hardening (elastic modulus = 200 GP, yield stress = 300 MPa, Poisson’s ratio = 0.3). The embedded-region constraint ensured full interaction between rebars and concrete. The loading level for both models was identical and was defined as a displacement-controlled load of 15 mm for a model length of 3 m. The concrete was discretized using C3D8R solid elements, while the longitudinal and transverse reinforcement were represented by 3D frame (B31) elements with six degrees of freedom per node. This approach captures the stress transfer mechanism while maintaining computational efficiency. Importantly, the boundary conditions were modeled as hinged at both ends to replicate experimental end-restraint conditions.

#### Static modeling (monotonic shear loading)

**Fig. 2:**
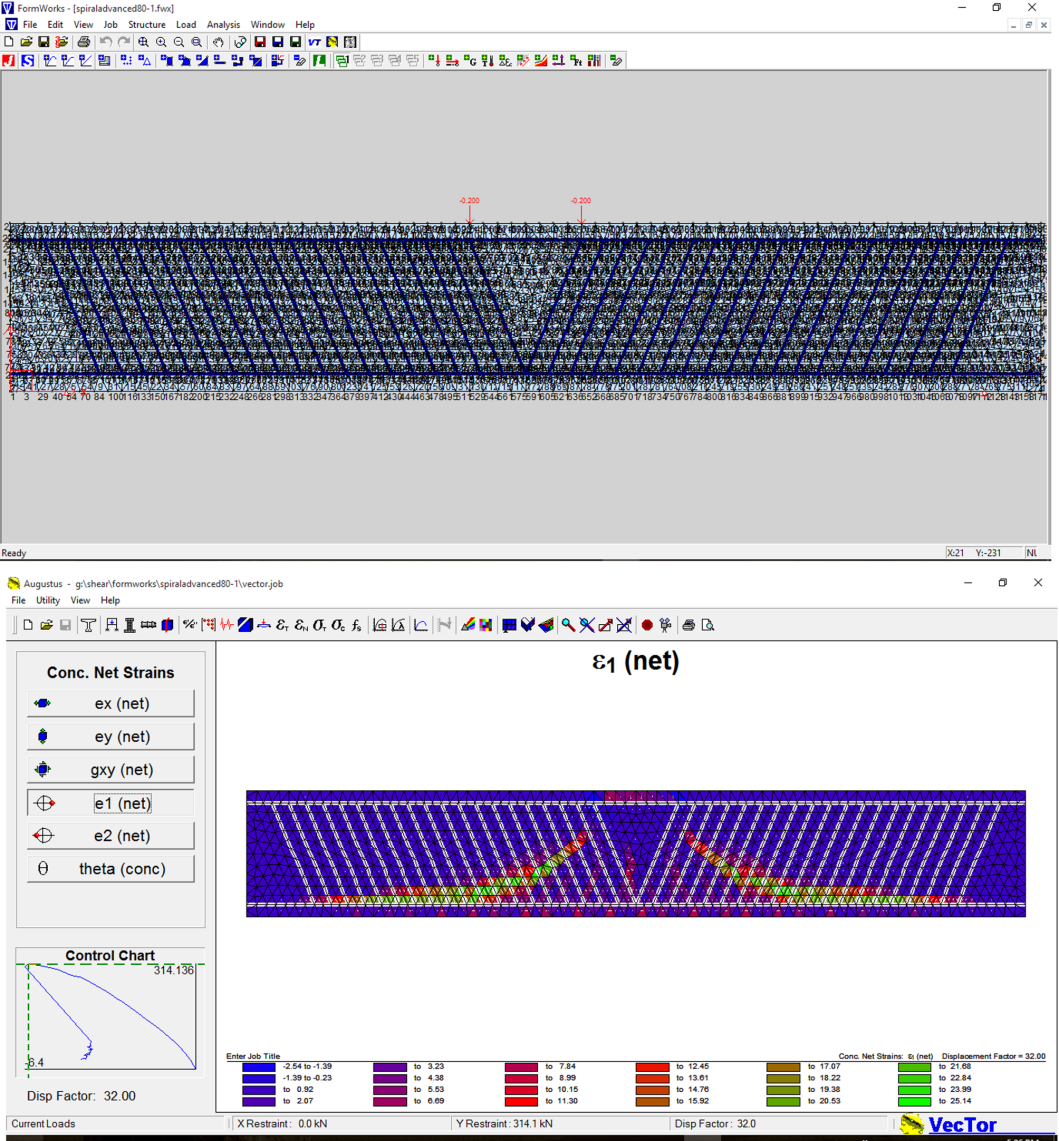
Pre-processor environment of VecTor2 showing the initial setup for modeling RC beams with RSR, based on the experimental configuration from Karayannis et al.^23^. This model setup was used for validation against experimental shear behavior before proceeding with our parametric analyses.

In recent years, the MCFT has been proposed as a basis for the analysis and design of two-dimensional reinforced concrete members by progressive design codes^47^ and reputable references^52–54^. In this section, the VecTor2 code and its pre-processor and post-processor programs (FormWorks and Augustus, respectively) are used to examine the shear capacity of beam members with continuous RSR and compare it with the capacity of similar members with conventional ties. This package provides a more accurate estimation of the structural performance (strength, post-peak behavior, failure mode, deformations, and cracking) of RC members compared to existing commercial tools. The theoretical basis of the VecTor2 program is composed of the MCFT and the DSFM. VecTor2 models cracked concrete as a rotating smeared-crack orthotropic material. This program uses an incremental iterative secant stiffness algorithm as an efficient and accurate numerical solution for solving problems. The modeling process began with replicating the experimental setup of Karayannis et al.^23^ in VecTor2’s pre-processor (Fig. 2), where we carefully matched material properties, boundary conditions, and reinforcement layouts. This preliminary validation step was crucial for ensuring our numerical approach could accurately reproduce experimental crack patterns before applying the methodology to our own specimens.

In this section, while validating the results of this research, other aspects of the RSR system, including the crack propagation in the models under shear and comparison of different methods (e.g., SMCFT, MPLANE, Vector2, CEB-FIP, CSA, AASHTO) in predicting the shear capacity of the samples are examined. For instance, MPLANE is a computational program developed at the University of Tokyo to simulate the in-plane behavior of RC panels using the LSFA. It models key mechanisms like crack formation, stress transfer between concrete and rebars, aggregate interlock, dowel action, and compression softening, serving as a virtual laboratory for RC panel analysis. Details of the other mentioned methods are provided in the authors’ previous study^39^.

#### Quasi-static modeling (lateral loading)

**Fig. 3:**
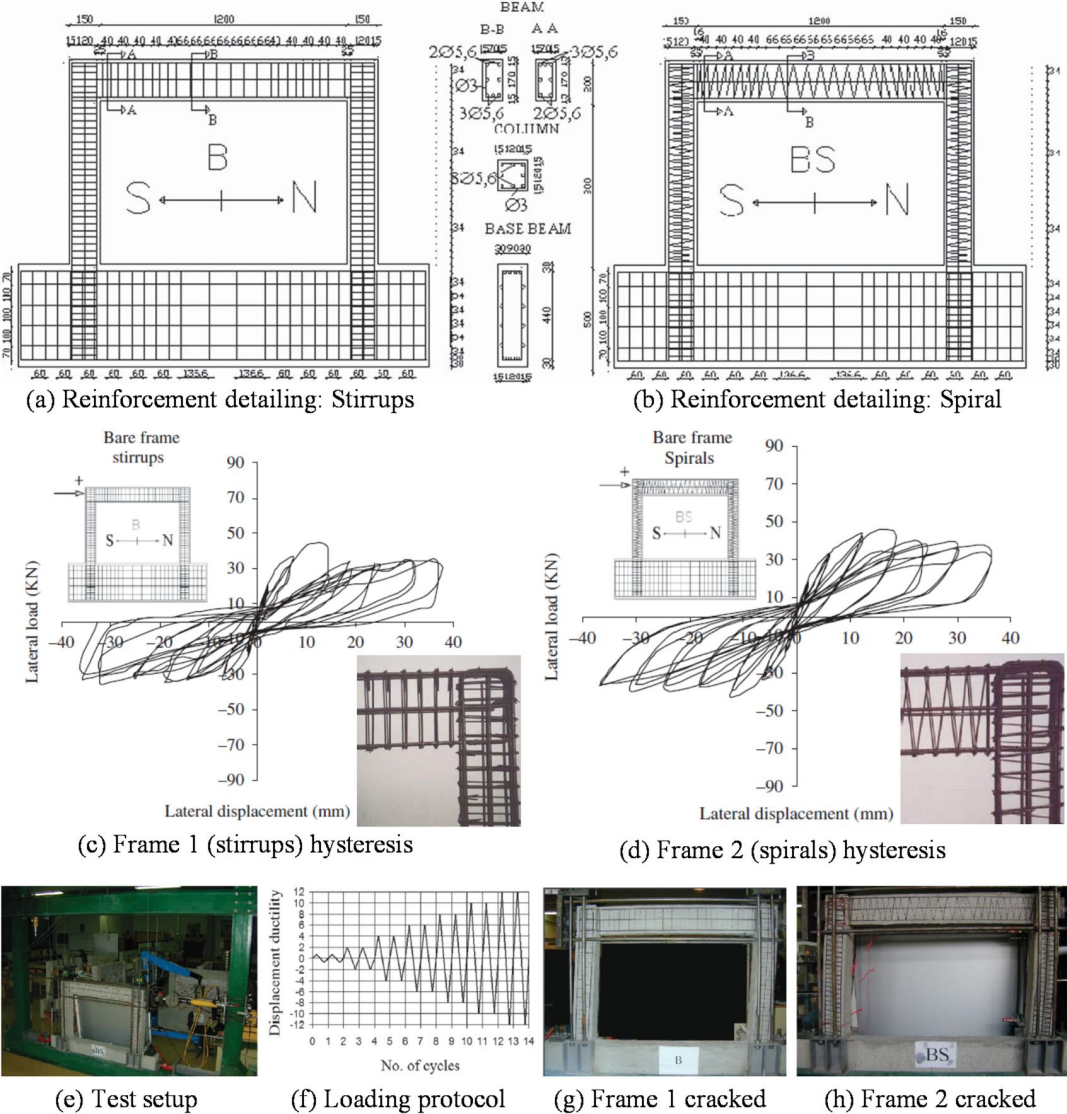
Specifications of the tested frame and its quasi-static response with conventional ties and RSR^55^.

To conduct a preliminary investigation into the effect of rectangular confinement on the envelope-based lateral quasi-static behavior of RC elements, the frame specimens (without infill walls) tested by Kakaletsis et al.^55^ were modeled and analyzed, as illustrated in Fig. 3a–h. The frame illustrated in Fig. 3a were reinforced with conventional transverse steel stirrups, whereas the specimen depicted in Fig. 3b incorporated a continuous RSR system, maintaining identical spacing in both configurations.

Both RC frame specimens exhibited identical geometric characteristics. Detailed elevation views, corresponding cross-sectional profiles of structural members, and reinforcement design specifications for the RC frames are presented in Fig. 3a,b. The beam and column cross-sectional dimensions measured 100 × 200 mm and 150 × 150 mm, respectively, representing a 1:3 scale reduction relative to the prototype frame sections, which featured beam and column dimensions of 300 × 600 mm and 450 × 450 mm. To mitigate brittle shear failure, each beam-column joint was reinforced with five closely spaced horizontal stirrups. Longitudinal reinforcement consisted of Ф5.60 mm diameter bars, while transverse reinforcement employed Ф3 mm diameter stirrups, corresponding to a 1:3 scale representation of the prototype frame’s Ф18 mm and Ф8 mm reinforcement, respectively. The transverse reinforcement detailing within the critical regions of the specimens complied with the stringent requirements stipulated by Greek seismic design codes. The mean compressive strength of the concrete used in the frame was 28.51 MPa. The yield stress values for the transverse and longitudinal steel reinforcement were 212.2 MPa and 390.47 MPa, respectively. These frames, constructed at a one-third scale (1.0 m × 1.5 m), were subjected to quasi-static lateral loading to evaluate quasi-static loop response and post-peak behavior. The resulting force-displacement curves are shown in Fig. 3c,d, indicating that the RSR-confined frame exhibited slightly improved capacity and post-peak performance compared to conventional ties. In this study, a numerical model was developed in SAP2000 to replicate these quasi-static loading scenarios, aiming to simulate the global structural response (e.g., lateral displacement, drift, and moment-rotation behavior) without explicitly modeling localized bond-slip mechanisms. The frame geometry, material properties, and loading protocol were implemented based on the specifications provided in Ref.^55^.

**Fig. 4:**
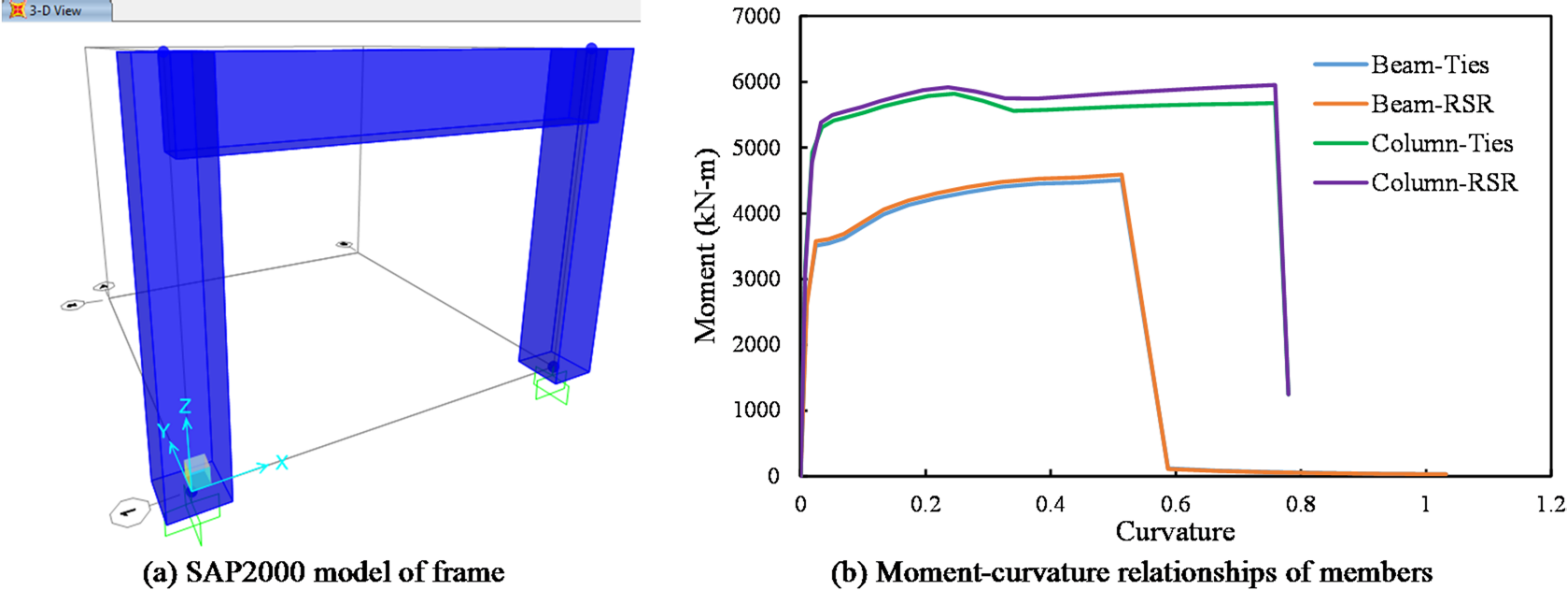
Input behavior of plastic hinges: moment-curvature relationships of members.

The Section Designer module was used to define the behavior of members and plastic hinges and the confined concrete model within the program environment was utilized to represent the behavior of the concrete core. To account for the effects of rectangular confinement in the model equipped with rectangular confinement, the amount of transverse reinforcement in the confined concrete model was adjusted using factor *η*_*RCTR*_ in Eq. (1).

1 $$\:\mathrm{R}\mathrm{e}\mathrm{s}\mathrm{p}\mathrm{o}\mathrm{n}\mathrm{s}\mathrm{e}\:=\:\mathrm{A}\:{tanh}\left(\mathrm{c}\:\mathrm{N}\mathrm{u}\mathrm{t}\mathrm{r}\mathrm{i}\mathrm{e}\mathrm{n}\mathrm{t}\:+\:\mathrm{b}\right)+\:\mathrm{B}$$

where η_RCTR_ is the rectangular confinement effectiveness coefficient that adjusts the transverse reinforcement effectiveness in confined concrete to reflect the geometry of rectangular sections. S′ is spacing of the transverse reinforcement, measured along the longitudinal axis of the column or beam. b_c_ and d_c_ are the center-to-center distance between longitudinal bars in the horizontal direction, representing the core width and length of the confined region. This equation was developed by the authors as a simplified method to incorporate rectangular confinement geometry into macro-scale numerical models (SAP2000). The conceptual basis of the formulation follows Mander et al. (1988)’s^49^ confined concrete model, extended to rectangular spiral configurations using an empirically calibrated confinement coefficient. The results are shown in Fig. 4 in terms of moment-curvature curves. By applying gravity loading to the structural model and imposing displacement constraints for lateral loading, the nonlinear behavior of the frames was analyzed.

#### Dynamic modeling (seismic loading)

Despite the many advantages of lateral quasi-static analysis, nonlinear time history seismic analysis is crucial for seismic design and retrofit^56,57^. Therefore, after the envelope-based lateral quasi-static validations, the full-scale frame with RSR confinement was investigated during earthquakes. For this 3D analysis, earthquake records from the FEMA-P695^58^ for site type II were used. The specifications of the three employed accelerograms are provided in Table 3. The accelerograms were scaled to simulate earthquakes in different levels of (a) operational, (b) moderate, (c) design, (d) maximum possible, and (e) very strong, corresponding to peak ground accelerations (PGA) of 0.06 g, 0.17 g, 0.35 g, 0.52 g, and 1.00 g, respectively.

**Table 3:** Specifications of the records used in the nonlinear analyses.

Event name	Station	*R*_rup_ (km)	Year	Magnitude	Component	PGA (g)	PGV (cm/s)
Cape mendocino	Petrolia	8.18 (near)	1992	7.01	090	0.66	88.51
Landers	Yermo Fire Station	23.62 (far)	1992	7.28	270	0.24	51.12
Manjil	Abbar	12.55 (far)	1990	7.37	L	0.51	42.46

### Testing program

This research experimentally evaluates the axial compressive performance of 14 RC short columns, organized into 7 test series. Each group includes one column transversely confined with identically spaced RSR and another reinforced with traditional ties. Table 4 outlines the characteristics of each sample.

**Table 4:** Specifications of specimens.

Test group	Symbol	Section dimensions (mm × mm)	Longitudinal reinforcement	Transverse reinforcement dimensions (mm × mm)	Transverse reinforcement type	Size of transverse reinforcement	S (mm)	f’’_c_ (MPa)	ρ_t_ (%)	ρ_l_ (%)	*N* (kN)	*P*_0_ (kN)
1	R4T8-72	250 × 180	4Ф12	220 × 150	Ties	Ф8	72	23.0	1.121	1.005	1256	1026
	R4C8-72	250 × 180	4Ф12	220 × 150	RSR	Ф8	72	23.0	1.130	1.005	1395	1026
2	S4T6-50	200 × 200	4Ф12	170 × 170	Ties	Ф6	50	26.4	1.128	1.131	1263	1043
	S4C6-50	200 × 200	4Ф12	170 × 170	RSR	Ф6	50	26.4	1.131	1.131	1465	1043
3	S4T6-50d	200 × 200	4Ф12	170 × 170	Ties	Ф6	50	24.9	1.128	1.131	1216	992
	S4C6-50d	200 × 200	4Ф12	170 × 170	RSR	Ф6	50	24.9	1.131	1.131	1394	992
4	S4T6-100	200 × 200	4Ф12	170 × 170	Ties	Ф6	100	25.8	0.565	1.131	1242	1022
	S4C6-100	200 × 200	4Ф12	170 × 170	RSR	Ф6	100	25.8	0.570	1.131	1295	1022
5	S4T8-75	200 × 200	4Ф12	170 × 170	Ties	Ф8	75	27.5	1.140	1.131	1349	1080
	S4C8-75	200 × 200	4Ф12	170 × 170	RSR	Ф8	75	27.5	1.149	1.131	1460	1080
6	S4T12-170	200 × 200	4Ф12	170 × 170	Ties	Ф12	170	24.4	1.128	1.131	1206	975
	S4C12-170	200 × 200	4Ф12	170 × 170	RSR	Ф12	170	24.4	1.131	1.131	1268	975
7	S8T6-85	200 × 200	8Ф8	170 × 170	Ties	Ф6	85	23.1	1.133	1.005	1242	917
	S8C6-85	200 × 200	8Ф8	170 × 170	RSR	Ф6	85	23.1	1.135	1.005	1236	917

According to this table, *H* (equal to 0.5 m in all tests) denotes the height of the sample, *S* represents the center-to-center spacing of the pitch of the spiral (ties), and *f’*_*c*_ indicates the characteristic strength of unconfined concrete regarding each test set. The volumetric ratios of longitudinal and transverse rebars are denoted as *ρ*_*l*_ and *ρ*_*t*_, respectively (excluding the length of bends and hooks). *N* refers to the load-carrying capacity. The parameter *P*_*0*_ represents the compressive strength of the samples based on ACI-CODE-318-19R22, calculated without accounting for accidental eccentricity or strength reduction factors. It is determined using the formula *P*_*0*_ *= 0.85 f’*_*c*_ (*A*_*g*_*-A*_*st*_) *+ f*_*yl*_*A*_*st*_, where *A*_*g*_ is the gross cross-sectional area for the column and *A*_*st*_ is the total area of longitudinal reinforcements. As shown in Table 4, the fixed parameter within each test set is *S*. The ρ_t_ values are slightly higher for samples incorporating RSR due to the inclination of continuous transverse reinforcement. However, when accounting for hook and bend lengths, it is important to note that, from an economic standpoint, RSR specimens generally use less reinforcement, as highlighted in^34^.

**Fig. 5:**
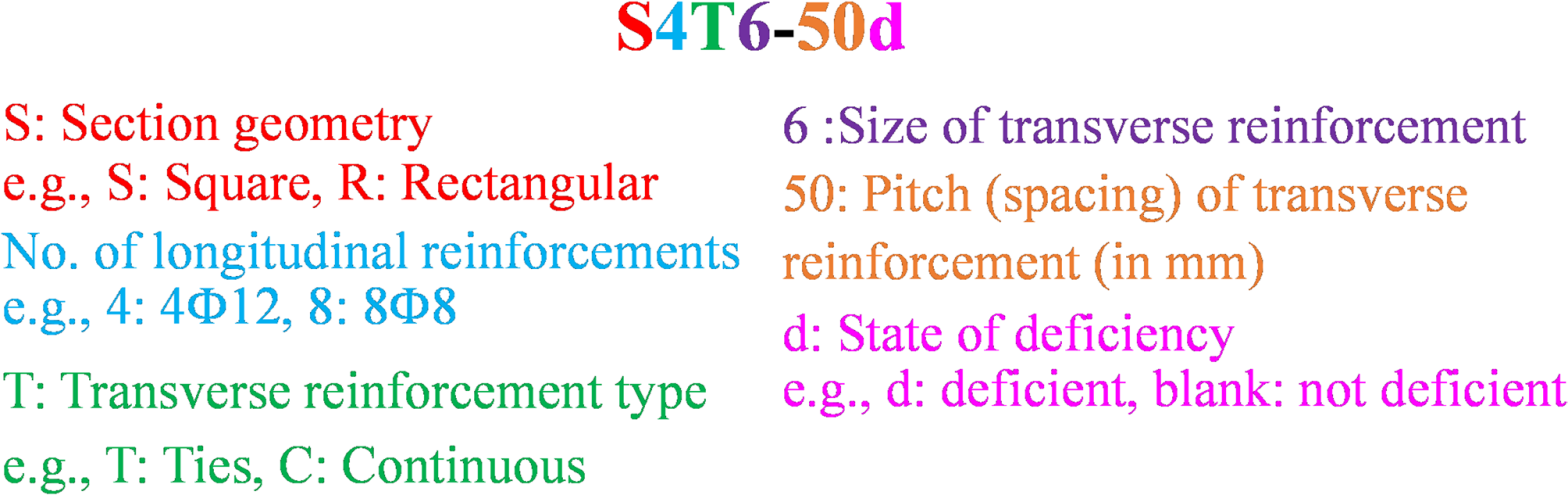
Specimen labeling system.

**Fig. 6:**
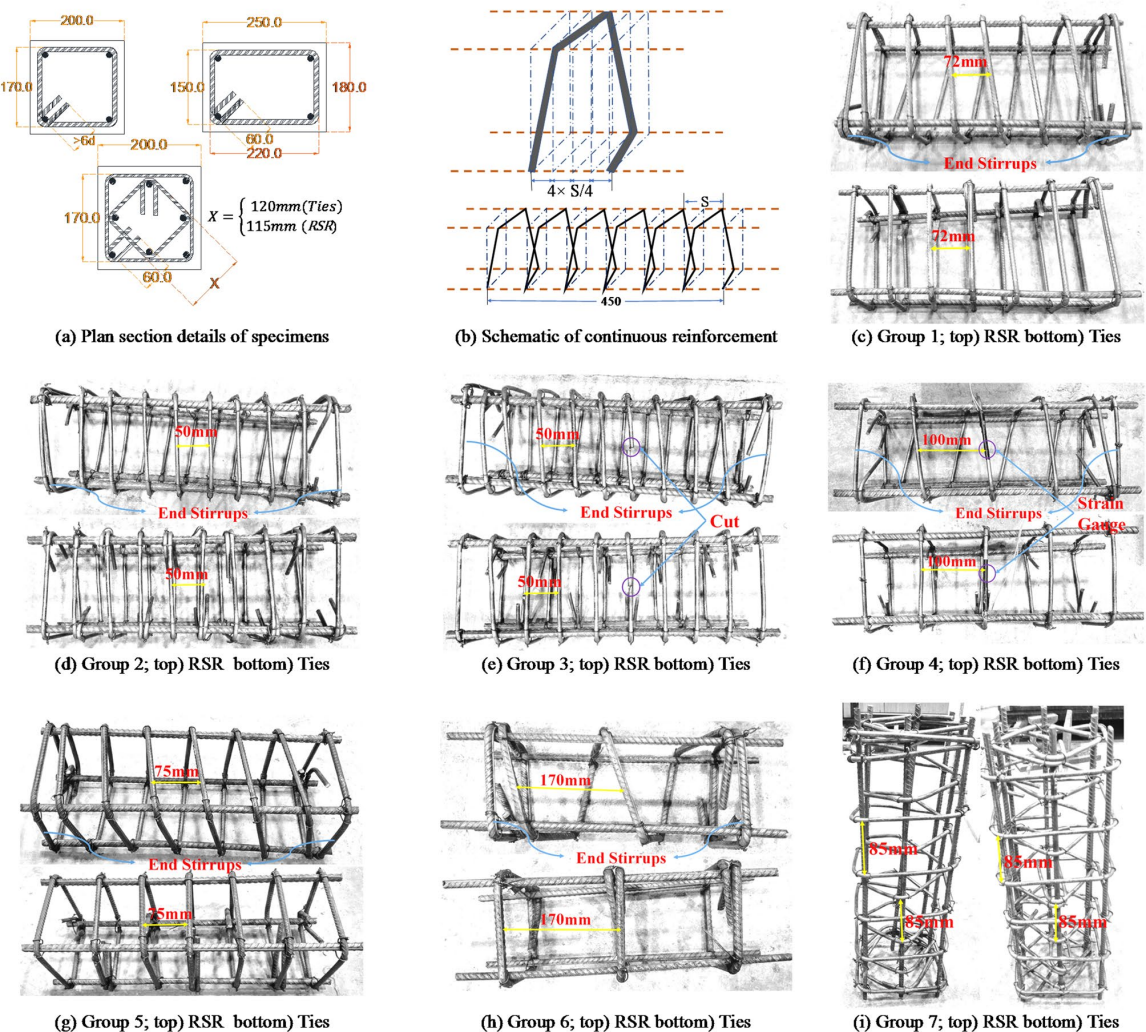
Reinforcement detailing.

The nomenclature of the specimens is meticulously defined in Fig. 5, with Fig. 6a–i detailing the reinforcement specifications. To accurately monitor the state of transverse reinforcement under varying load levels, a strain gauge was strategically placed in each element of the test group 4 samples, as depicted in Fig. 6f.

The mechanical properties of the rebars are presented in Table 5. Here, *f*_*y*_ and *f*_*u*_ denote the yield and ultimate stresses, respectively. The strain at yield, ultimate strength, and strain hardening are represented by *ε*_*y*_, *ε*_*u*_, *ε*_*sh*_, respectively. These parameters are crucial in understanding the load-bearing contribution of the rebars in the RC column samples.

**Table 5:** Properties of reinforcing bars.

Reinforcement	Ф12	Ф8	Ф6
Type	Ribbed	Ribbed	Smooth
Diameter (mm)	12	8	6.5
ε_y_	0.00171	0.00173	0.00166
ε_u_	0.16500	0.13800	0.09700
ε_sh_	0.01534	0.02458	0.00450
*f*_*y*_ (MPa)	343	347	338
*f*_*u*_ (MPa)	530	487	499
Elongation (%)	30.1	22.7	18.6

**Fig. 7:**
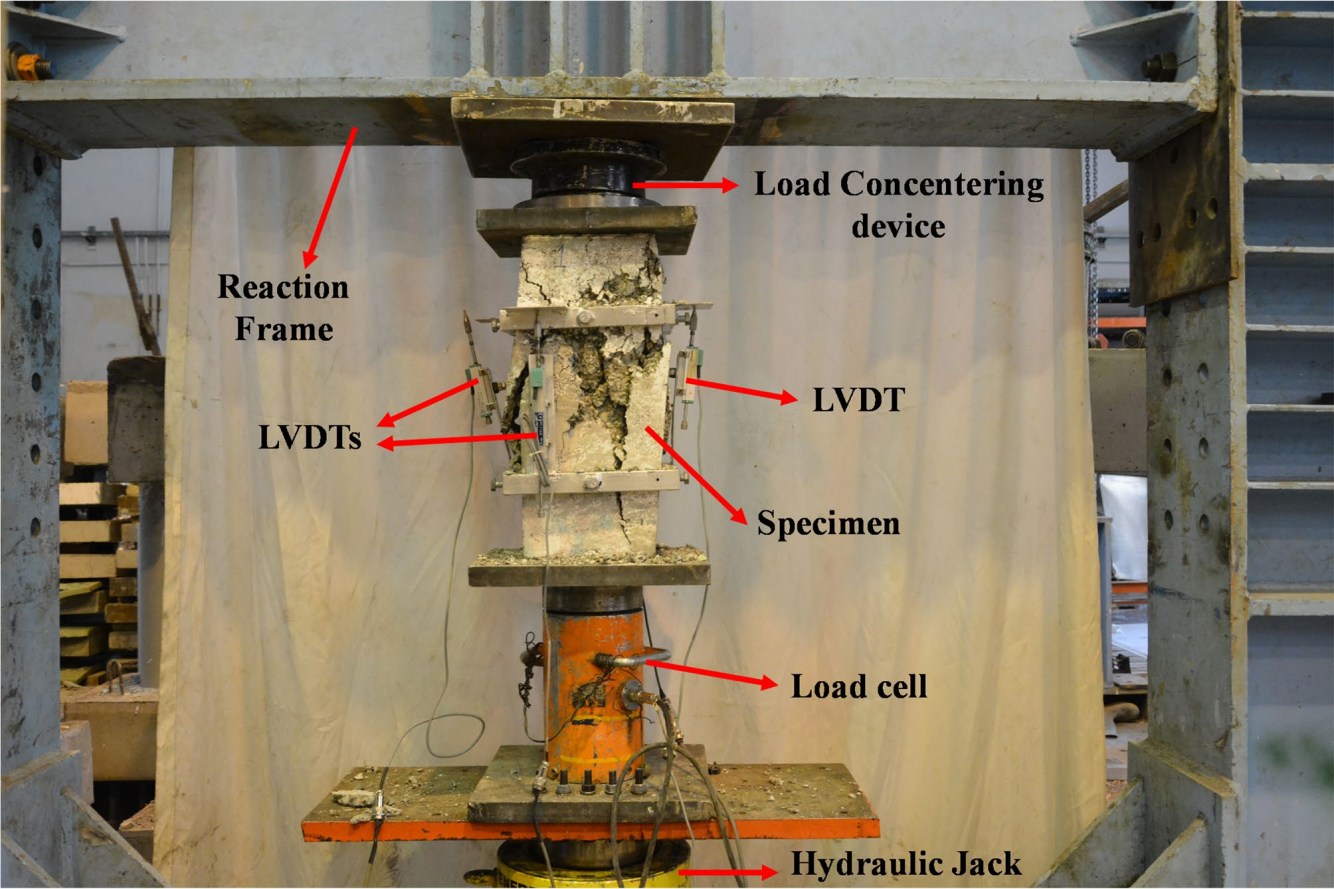
Test instrumentation.

Figure 7 showcases the test setup and instrumentation.

The loading was applied under displacement-controlled conditions using a servo-controlled hydraulic jack (rated for a lifting capacity of 3 MN) capable of maintaining a highly stable and accurate loading rate. The system was calibrated to achieve a strain rate of approximately 10^−5^ per second, a standard rate recommended for quasi-static axial compression testing of concrete members. Load measurements were taken using a robust 3 MN load cell, while compressive deformation was meticulously recorded using four LVDTs with an exceptional accuracy of 10^−6^ m, spanning a 260 mm length in the middle of the samples. The jack’s displacement control was continuously monitored by both the built-in LVDT and the external LVDTs fixed at mid-height of the column. The data were then averaged to ensure precision in the results. To avert premature asymmetrical failure of the specimens under compression, a custom-engineered load-centering device, featuring a convex-concave assembly, was employed as illustrated in Fig. 7. This setup was essential for obtaining reliable and consistent experimental results.

### Mander et al.^49^ model and proposed analytical model

**Fig. 8:**
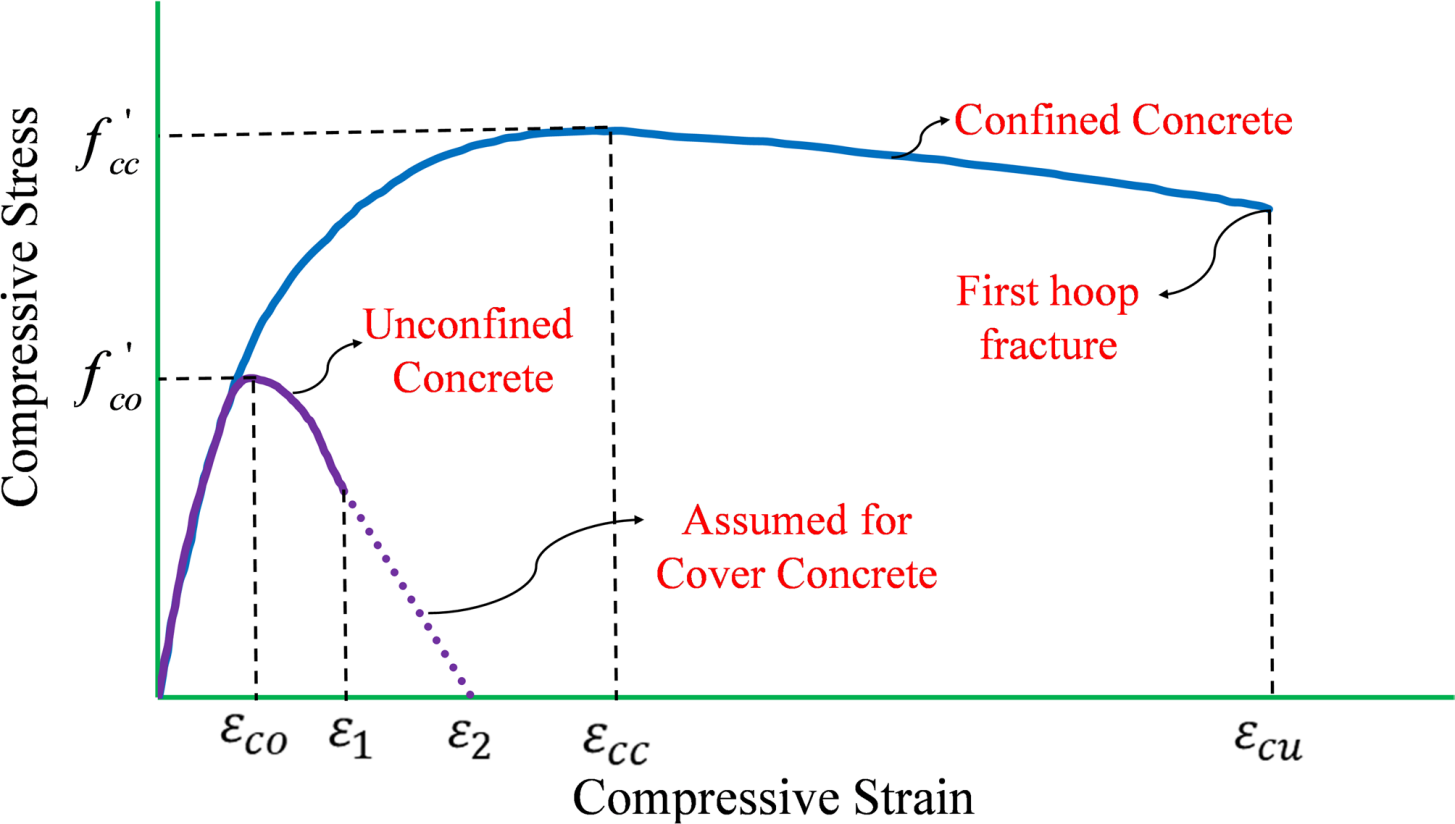
Mander’s model^49^ for unconfined and confined concrete.

Analytical models serve as foundational tools for developing numerical solutions to complex engineering problems. Their defined characteristics, such as accuracy, computational efficiency, simplicity, and cost-effectiveness, establish valuable benchmarks, especially when experimental data is unavailable^59^. In this section, a novel theoretical model is presented for the precise prediction of the compressive performance of RC columns incorporating RSR. The model emphasizes the critical role of three key components: the longitudinal rebar, the cover concrete, and the core concrete. To develop a comprehensive and versatile model for the entire member, a deep and thorough understanding of each component’s behavior is crucial. Mander et al.^49^ proposed a highly regarded stress-strain model for confined and unconfined concrete (Fig. 8).

While the original equations of the Mander’s model are well-known and widely documented, only the final governing formulae used in the current study are presented below. For reference, the complete derivation and intermediate expressions from the original model are provided in Appendix A and Table A1.

The proposed modifications incorporate a refined effectiveness coefficient *k*_*e*_ ​ specific to RSR geometry, calibrated against experimental data (see Fig. 25), and account for the inclined path of RSR that leads to improved lateral confinement uniformity. The spalling thresholds ε_*1*_​ and ε_2_ were selected based on test observations rather than assumed constants. These refinements ensure that the model accurately reflects the stress–strain response of RSR-confined RC columns.

**Fig. 9:**
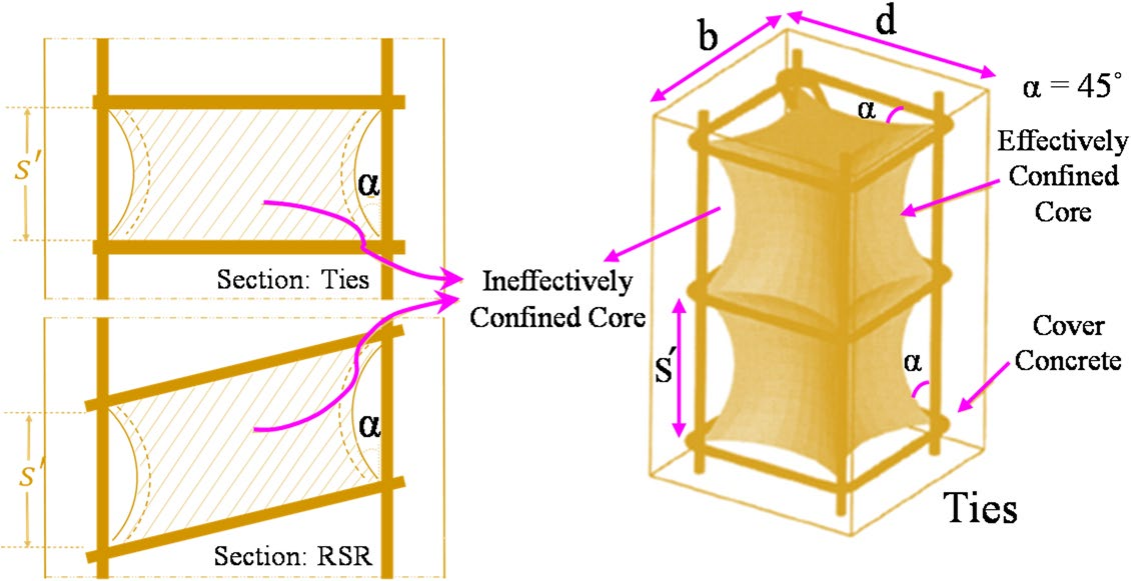
Arch action in compressive performance of RC elements with discrete and continuous lateral reinforcements^60^ (3D image regenerated using Google Gemini, version 2.5 pro, https://gemini.google.com/).

According to the concept of arch action (discussed in^49^), as shown in Fig. 9, the aforementioned method can be extended theoretically for RSR. The following formula (Eq. 2) is proposed for confinement effectiveness coefficient, *k*_*e*_ of concrete cores with RSR:

2 $$k_{e} = \frac{{\left( {1 - \sum {\frac{{\left( {w_{i}^{'} } \right)^{2} }}{{6b_{c} d_{c} }}} } \right)\left( {1 - \frac{{s^{'} }}{{4b_{c} }}} \right)\left( {1 - \frac{{s^{'} }}{{4d_{c} }}} \right)}}{{\left( {1 - \rho _{{cc}} } \right)}}$$

**Fig. 10:**
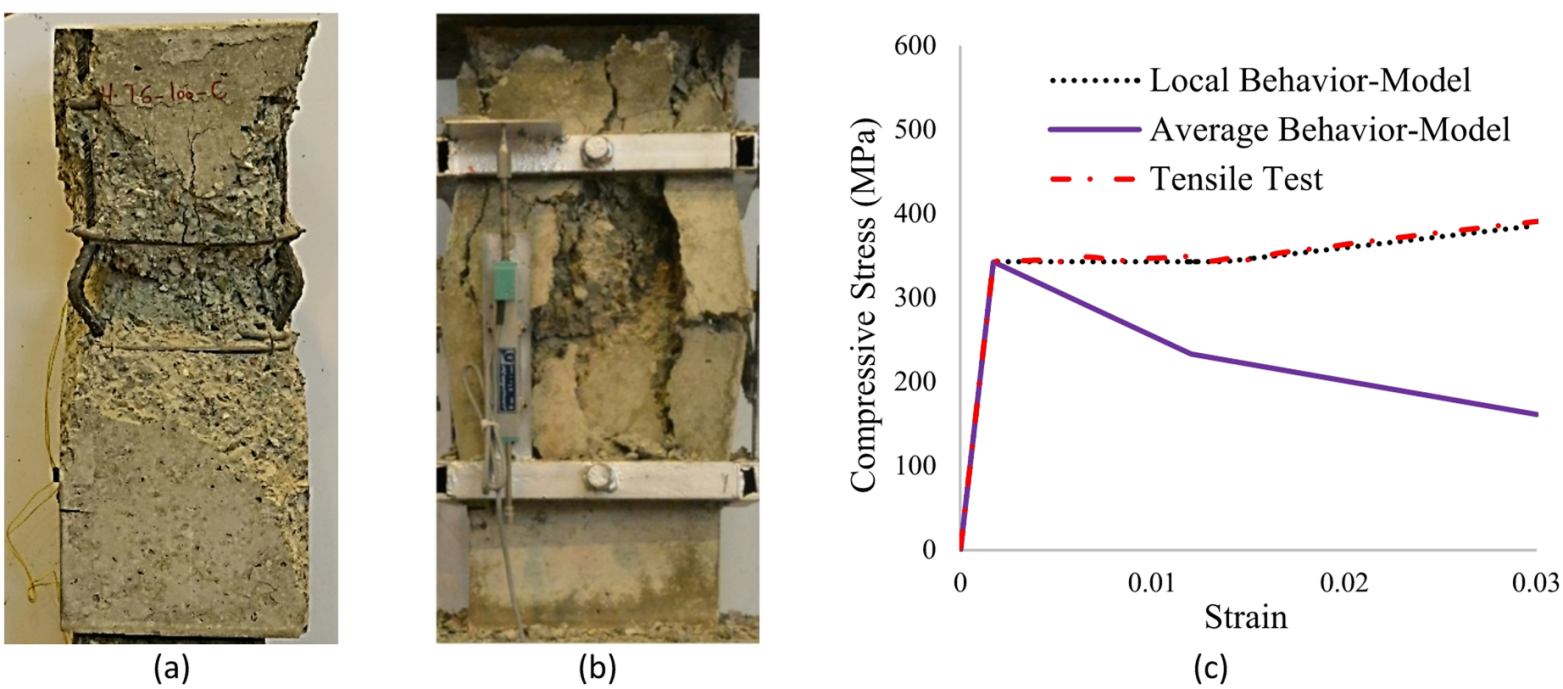
Severe spalling of the cover concrete and significant rebars buckling in sample S4T12-170. (**a**) Longitudinal rebars’ buckling, (**b**) Cover concrete spalling, (**c**) Model of Dhakal and Maekawa^61^ buckling.

A significant incident that occurs in RC members under high compression is the spalling of cover concrete, as shown in Fig. 10b. Mander’s stress-strain model^49^, illustrated in Fig. 8, uses a two-portion curve to represent the performance of cover concrete. The 1st portion is according to the unconfined concrete model, while the linear 2nd portion, aims to simulate the impacts of the spalling phenomenon. Mander’s model uses two key points for spalling; *ε*_*1*_ *= ε*_*co*_ corresponding to the initiation of spalling and *ε*_*2*_ *= ε*_*sp*_ which corresponds to the completion of spalling. In this study, a similar approach was used with the difference that instead of prescribed values, *ε*_*1*_ and *ε*_*2*_ were chosen in accordance with accurate observations.

Another critical issue affecting the performance of RC elements during compression loading is the buckling of longitudinal reinforcements (Fig. 10a), which can profoundly impact post-peak performance. Dhakal and Maekawa^61^, through rigorous analytical and numerical studies, developed a robust model for the axial behavior of reinforcements. This model has demonstrated excellent agreement with experimental results, particularly in scenarios where reinforcement buckling is a significant factor. The model’s formulation for monotonic compression is presented in Eqs. (3)–(6).

$$\frac{\sigma }{{\sigma _{l} }} = 1 - \left( {1 - \frac{{\sigma ^{*} }}{{\sigma _{l}^{*} }}} \right)\left( {\frac{{\varepsilon - \varepsilon _{y} }}{{\varepsilon ^{*} - \varepsilon _{y} }}} \right)\,{\mathrm{for}}\,\varepsilon _{y} < \varepsilon \le \varepsilon ^{*}$$

3 $$\sigma \ge 0.2f_{y} ;\sigma = \sigma ^{*} - 0.02E_{s} \left( {\varepsilon - \varepsilon ^{*} } \right)\,{\mathrm{for}}\,\varepsilon > \varepsilon ^{*}$$

4 $$\frac{{\varepsilon ^{*} }}{{\varepsilon _{y} }} = 55 - 2.3\sqrt {\frac{{f_{y} }}{{100}}} \frac{L}{D};\frac{{\varepsilon ^{*} }}{{\varepsilon _{y} }} \ge 7$$

5 $$\frac{{\sigma ^{*} }}{{\sigma _{l}^{*} }} = \alpha \left( {1.1 - 0.016\sqrt {\frac{{f_{y} }}{{100}}} \frac{L}{D}} \right);\sigma ^{*} \ge 0.2f_{y}$$

6 $$\begin{gathered} \alpha = 0.75 + \frac{{\varepsilon _{u} - \varepsilon _{{sh}} }}{{300\varepsilon _{y} }} \hfill \\ \alpha \le \frac{{f_{u} }}{{1.5f_{y} }},0.75 \le \alpha \le 1.0 \hfill \\ \end{gathered}$$

where *D* is the rebar diameter, *E*_*s*_ is the modulus of elasticity of the rebar, *L* is the length of buckling, *σ* and *ε* represent the average stress and strain, σ* and ε^*^ denote the stress and strain at an intermediate point, and *σ*_*l*_ and *σ*_l_* are the local stresses at the current and ε^*^ strains, respectively. The remaining parameters have been previously defined. For a vivid illustration, Fig. 10c showcases the behavior of longitudinal rebars for specimen S4T12-170, as modeled by Dhakal and Maekawa^61^. The figure presents three key components of Dhakal and Maekawa^61^ buckling model as applied to our specimen S4T12-170. Local Behavior represents the local compressive stress-strain response at any location of steel bars, showing yield point at ε_y_ = 0.0017 (343 MPa) and significant average stress reduction due to buckling. Average Behavior Shows the member-level response accounting for buckling along bar length and interactive effects between concrete confinement and steel yielding. Tensile Test Reference provides baseline monotonic tensile behavior for comparison. The Model of Dhakal and Maekawa^61^ buckling captures three distinct phases observed in our tests:

1. Pre-buckling: Linear elastic behavior matching tensile properties.
2. Buckling initiation:

- 15% stiffness reduction due to geometric nonlinearity.
- Onset of visible buckling at L/170 slenderness ratio.

3. Post-buckling:

- More than 22% load capacity drop versus tensile reference.
- Progressive localization of deformations.

## Results

### Preliminary numerical results

Preliminary results are presented based on the model described in “Preliminary prediction of confinement with RSR based on a simple numerical model”.

#### Static behavior of RC elements with RSR under compression loading

**Fig. 11:**
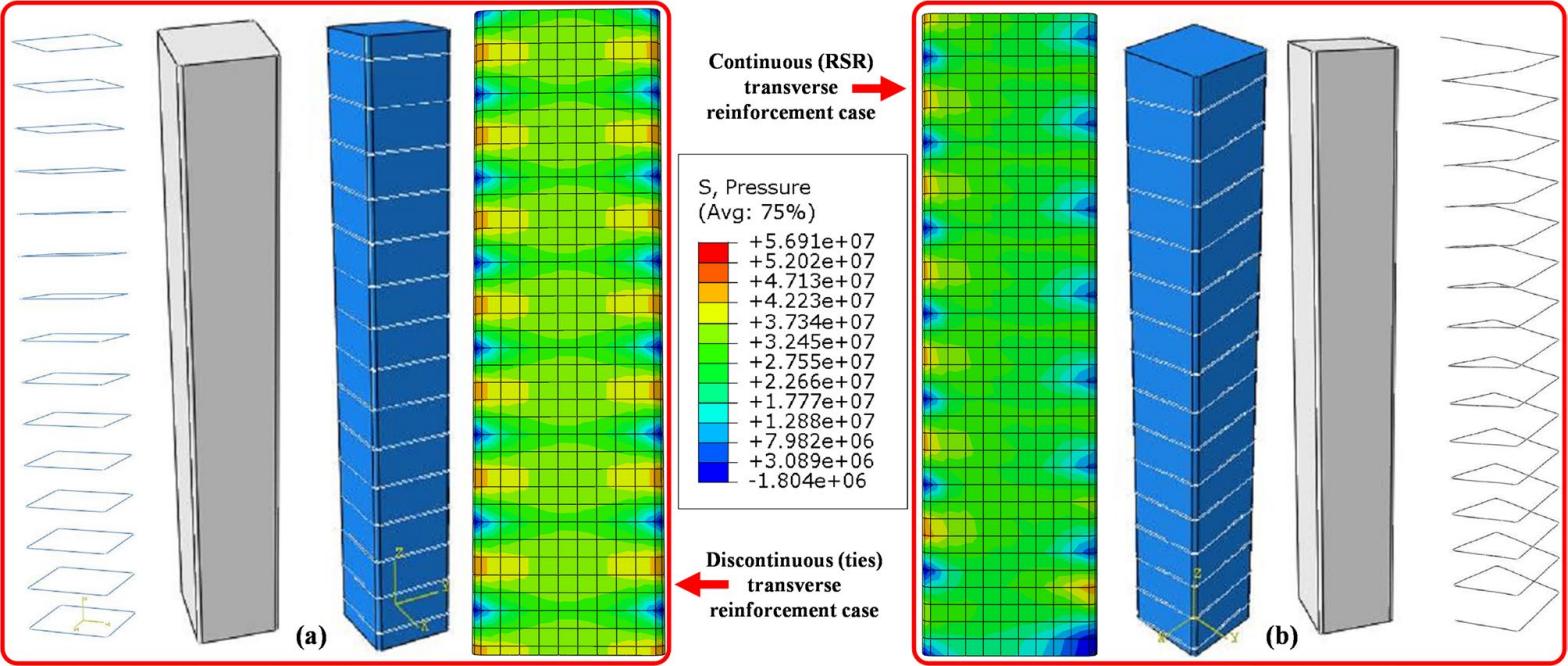
Nonlinear finite element results and hydrostatic pressure stress distribution in the square concrete core with transverse reinforcements under axial load: (**a**) conventional; (**b**) and continuous (Generated using ABAQUS software, version 6.14-2, Dassault Systèmes, https://www.3ds.com/products-services/simulia/products/abaqus/).

This section discusses compressive behavior using the model explained in “Static modeling (monotonic compression loading)”. By comparing the compressive stress state in the core concrete body in two models as shown in Fig. 11a,b, it can be observed that the assumption of the arching action is largely consistent with reality.

The hydrostatic pressure stress (S) in Fig. 11 is computed as the average of the three normal stresses (σ_1_, σ_2_, σ_3_) acting on a point in a three-dimensional body: S = (σ_1_ + σ_2_ + σ_3_)/3. The nonlinear analysis confirms that the RSR-confined concrete exhibited a smoother transition to the inelastic regime. The stress concentration is clearly higher in the sample with discontinuous transverse reinforcement, while the concrete core equipped with spiral reinforcement shows a more uniform stress distribution. The promising results from these cases were utilized to conduct further tests under various conditions of the RSR system. The observed uniformity of stress distribution in the RSR-confinement model demonstrated strong correlation with established experimental trends, thereby validating the appropriateness of the simplified analytical approach while confirming that the comparative assessment of confinement effectiveness between tie and RSR systems was sufficiently robust to justify subsequent experimental validation.

#### Static behavior of RC elements with RSR under shear loading

**Fig. 12:**
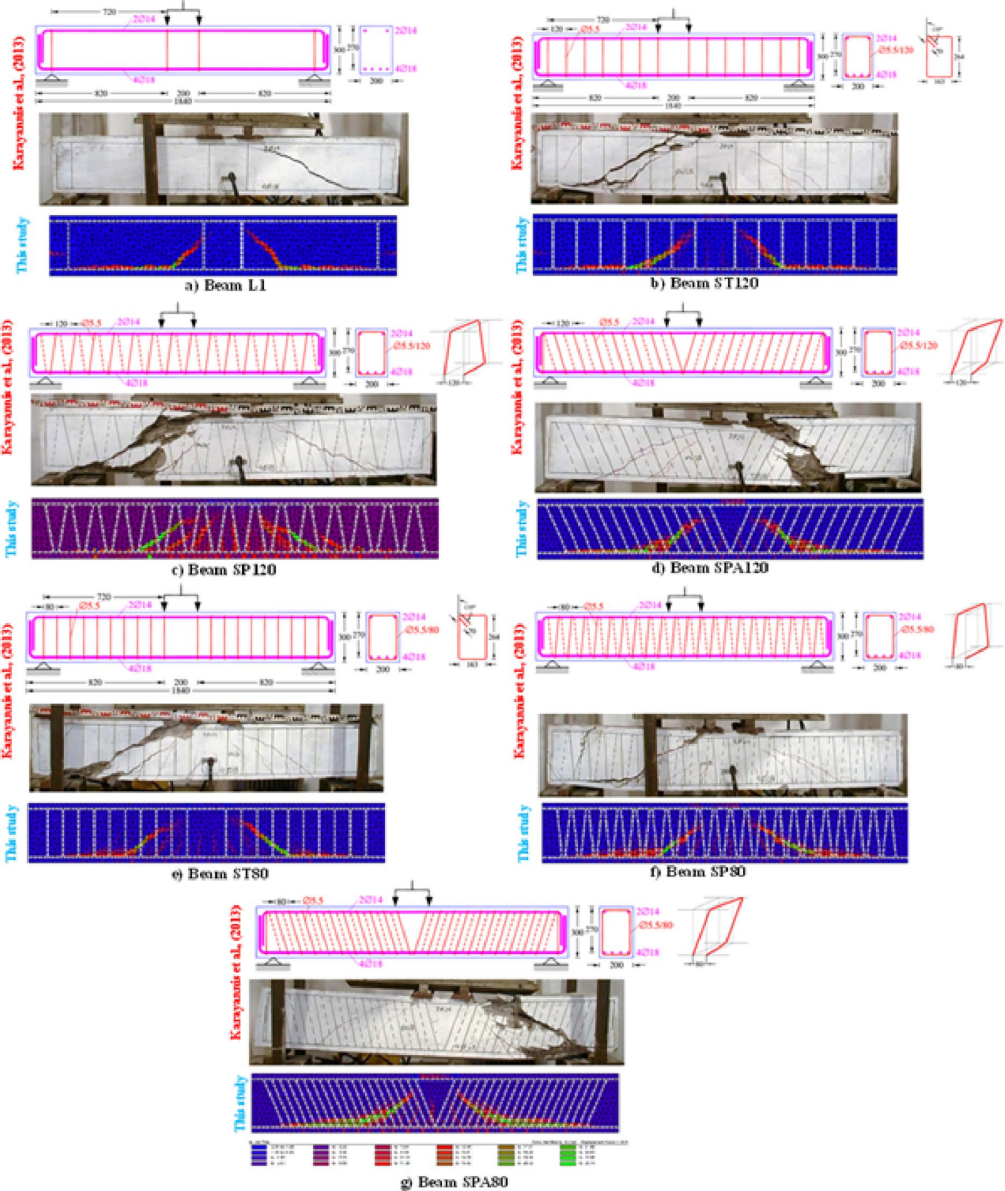
Comparison of the crack pattern between laboratory samples^23^ and VecTor2 numerical model.

The shear analysis is based on the modeling approach in “Static modeling (monotonic shear loading)”. Figure 12 shows the comparison of the diagonal crack pattern obtained from the nonlinear finite element analysis using VecTor2 with the results obtained from the tests. The validation process initiated in Fig. 1 yielded the crack pattern comparisons shown in Fig. 12, demonstrating excellent agreement between our numerical models and experimental results for both conventional and RSR-reinforced beams. By comparing Fig. 12a with Fig. 12b–g, it can be observed that the use of stirrups changes the crack inclination angle. However, no significant difference in the cracking angle between samples confined with ties and RSR was observed. Instead, there was a slight difference in the crack propagation pattern in samples confined with RSR compared to those confined with ties and more blunt crack patterns were developed in the RSR cases (Fig. 12b vs. Fig. 12c,d and Fig. 12e vs. Fig. 12f,g). Although some differences in crack inclination angles exist between the VecTor2 model and the experimental observations, these are primarily due to simplified boundary conditions and idealized material properties. VecTor2 models cracking using a smeared rotating crack theory, which tends to smooth out localized effects. Nevertheless, the overall location, extent, and density of cracks, as well as the failure mechanisms, show good agreement with the experiments, validating the model’s reliability for shear capacity prediction. Subsequently, using the methods presented in the authors’ previous study^39^, the shear capacity of the samples tested in reference^23^ has been calculated. The results of these calculations and analyses are reported in Table 6. The shear capacities reported in this table were calculated using a combination of nonlinear finite element analysis (VecTor2, MPLANE), analytical design codes (SMCFT, AASHTO, CSA, CEB-FIP), and validated experimental results from literature^23^. Input parameters for all models were derived from experimental setups and matched to specimen properties. For each method, the predicted shear capacities were computed using their respective constitutive models or code-based equations.

The following conclusions can be drawn:

- For the sample without transverse reinforcement, methods based on SMCFT significantly underestimate the shear capacity of the beam, while the more accurate methods VecTor2 and MPLANE overestimate this value compared to the experimental results.
- For the samples with transverse reinforcement, almost all values obtained from analytical and computational methods (except for the SP120 sample) are considerably higher than the experimental values. For methods based on SMCFT, it is expected that this trend should be reversed. A possible explanation for this issue is the unreliability of the reported material properties, especially *f’*_*c*_.
- According to the experimental results, using RSR leads to a 15% improvement in the shear capacity of the beam compared to the sample with conventional ties. As shown in Table 6, the SMCFT-based methods and their corresponding codes not only fail to predict this increase but also estimate a lower capacity than the case with conventional stirrups.
- The MPLANE and VecTor2 programs report some improvement in shear capacity for members with continuous RSR compared to conventional types, but this improvement is less than the actual value.
- The improvement due to the special continuous stirrups is predicted by all methods. This improvement is mainly due to the increased contribution of the reinforcement to the shear capacity.

**Fig. 13:**
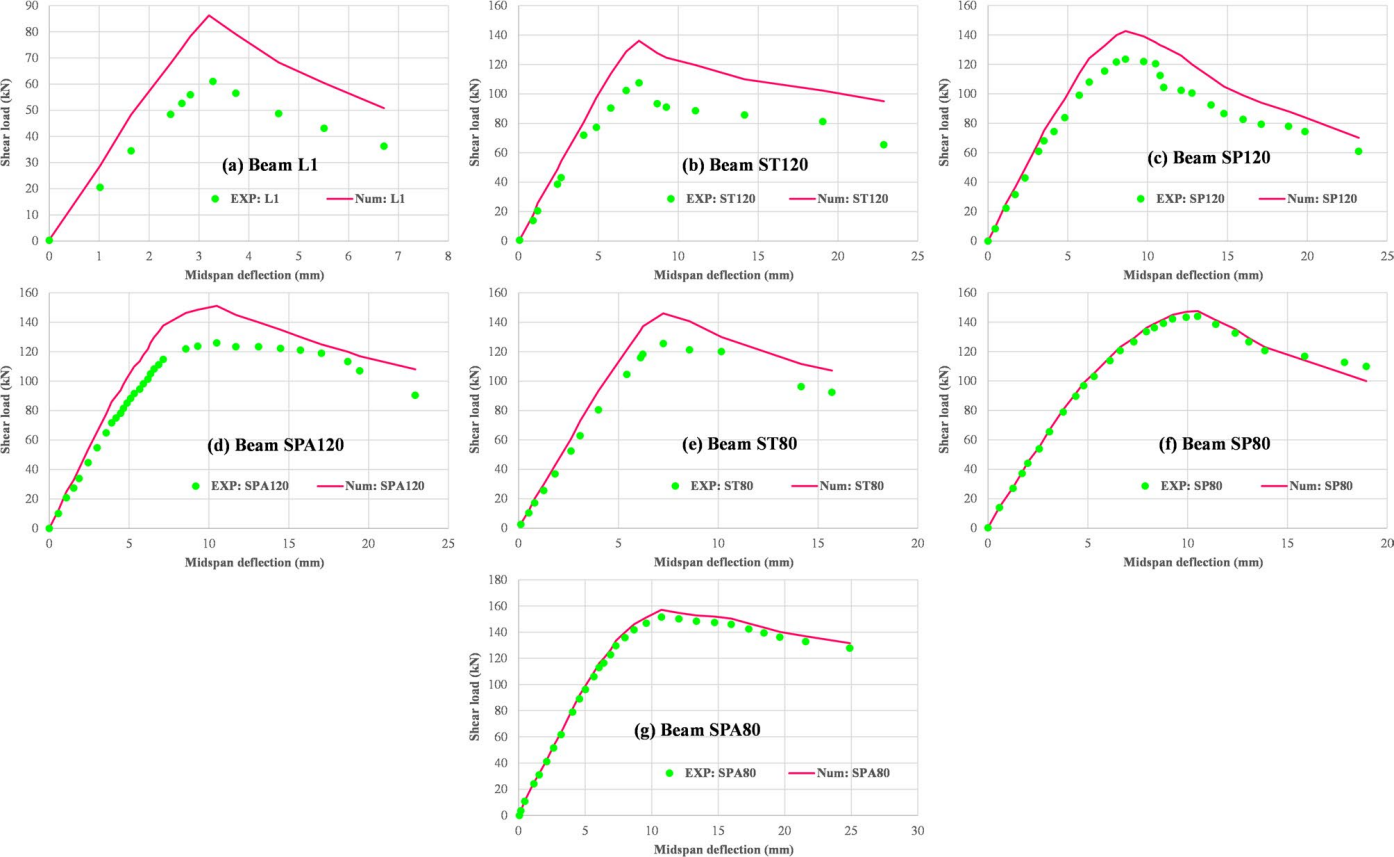
Comparison of the load–deflection behavior between the test results^23^ and VecTor2 numerical model.

**Fig. 14:**
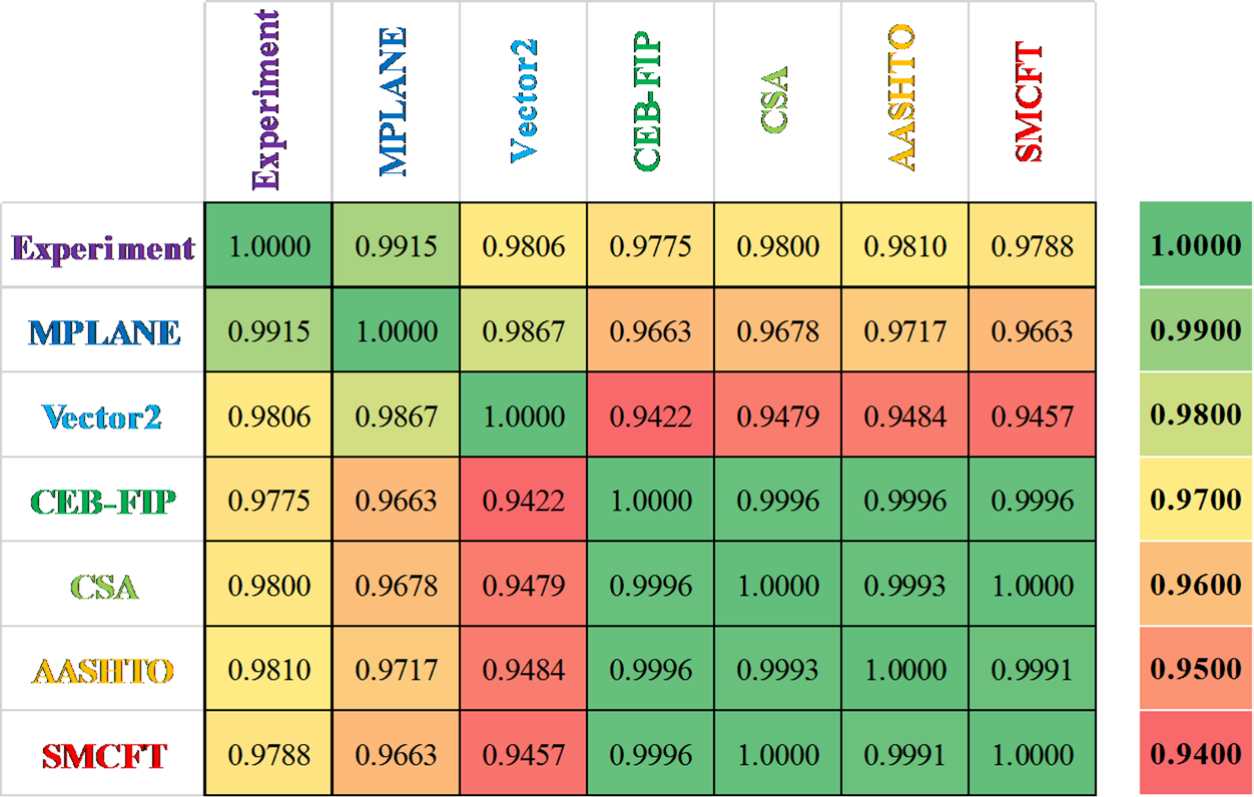
Spearman’s correlation matrix of shear capacity of beam samples from different methods.

Figure 13a–g presents a direct comparison between the experimental load-deflection response of the tested beam specimens and the results predicted by the nonlinear finite element analysis using VecTor2. As shown, the numerical model demonstrates a close correlation with the experimental behavior in terms of initial stiffness, peak strength, and post-peak behavior. Although some divergence is noted in the softening region beyond peak load—due to the simplified material models and the lack of bond-slip simulation—the overall agreement validates the reliability of the modeling approach. This result confirms the suitability of VecTor2 and the underlying MCFT and DSFM framework for simulating the behavior of RC beams with RSR under shear loading conditions. To evaluate the consistency and reliability of the results, the shear capacities of various beam samples from each method were analyzed, and Spearman’s correlation coefficients (R^2^) were calculated for each pair of methods. As shown in Fig. 14, these coefficients indicate a strong correlation among the methods, with values exceeding 94%, suggesting high reliability. Further analysis highlighted a particularly strong correlation among the CEB-FIP, CSA, AASHTO, and SMCFT methods, setting them apart from Vector2, MPLANE, and the experimental results. This finding highlights the versatility and potential applicability of newer approaches, such as Vector2 and MPLANE, in shear prediction scenarios. However, the sensitivity analysis revealed that the MPLANE method, in particular, demonstrates exceptional reliability, with a correlation coefficient of 99% when compared to experimental data.

**Table 6:** Comparison of shear capacity of beam samples obtained from different methods.

Approach	Experiment	MPLANE^44^	Vector2^45^	CEB-FIP^46^	CSA^47^	AASHTO^48^	SMCFT^43^
L1 (kN)	61.000	84.500	86.300	45.760	44.390	48.884	44.390
ST120 (kN)	107.500	128.790	136.100	122.430	121.220	118.620	124.480
SP120 (kN)	123.500	133.890	142.635	122.100	120.940	118.360	124.190
SPA120 (kN)	126.000	135.600	151.100	136.090	134.830	132.440	137.890
SP120/ST120	1.149	1.040	1.048	0.997	0.998	0.998	0.998
SPA120/ST120	1.172	1.053	1.110	1.112	1.112	1.117	1.108
ST80 (kN)	125.500	149.680	146.000	160.060	153.770	154.260	158.600
SP80 (kN)	144.000	162.320	147.510	159.590	153.360	153.860	158.170
SPA80 (kN)	151.500	166.210	157.070	180.220	174.330	174.970	178.870
SP80/ST80	1.147	1.084	1.010	0.997	0.997	0.997	0.997
SPA80/ST80	1.207	1.110	1.076	1.126	1.134	1.134	1.128

#### Quasi-static behavior of RC elements with RSR under lateral loading

**Fig. 15:**
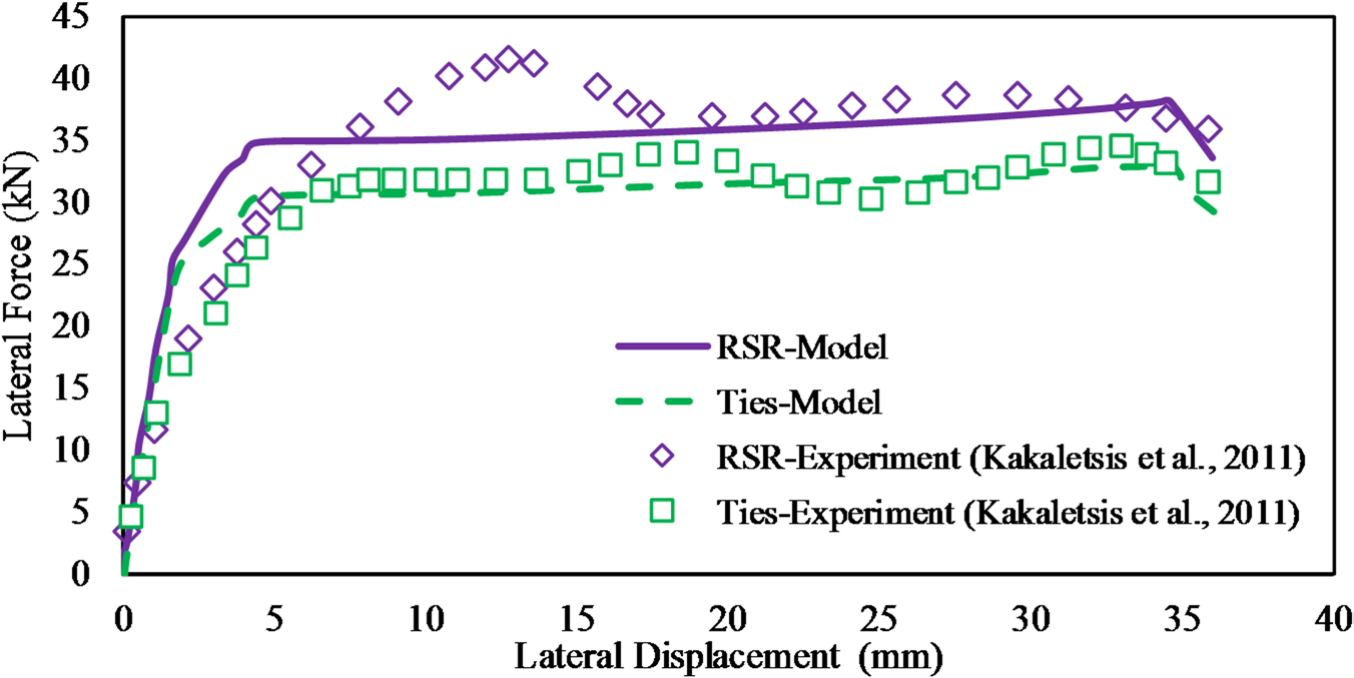
Comparison of the lateral behavior envelope curves from experimental^55^ and numerical models.

The lateral quasi-static behavior is examined using the method outlined in “Quasi-static modeling (lateral loading)”. Although this section references results from quasi-static analyses, it should be emphasized that the present study did not simulate full cyclic loading. The discussion herein refers to the envelope response under lateral static loads, which provides an approximate indicator of lateral stiffness and strength, not true cyclic or hysteretic behavior. The lateral load behavior envelope curves of the models were compared with the experimental behavior (by Kakaletsis et al.^55^) as shown in Fig. 15. It is observed that the proposed structural model predicts some performance improvement due to the RSR confinement. This improvement results from the better confinement effect of the RSR, which was anticipated to have a limited impact on flexural behavior. While commercial software like SAP2000 has limitations in fully capturing the complex hysteretic behavior of RC frames (including pinching effects and stiffness degradation), the envelope curves presented in Fig. 15 provide a reasonable approximation of the lateral quasi-static performance. The close agreement between experimental and numerical envelope curves suggests that the model captures the key aspects of strength and deformation capacity, which are critical for seismic assessment.

#### Dynamic behavior of RC elements with RSR under seismic loading

**Fig. 16:**
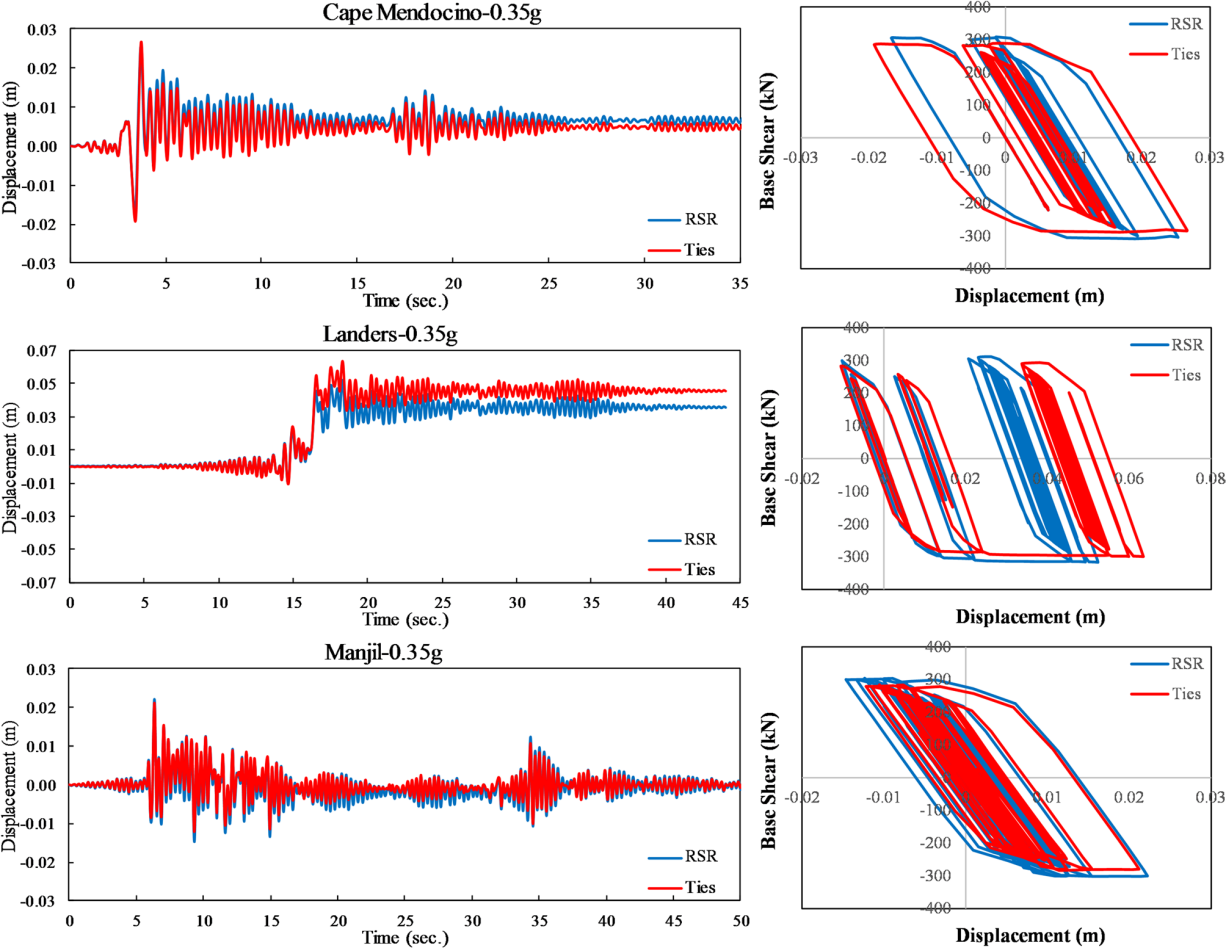
Comparison of roof displacement time history and base shear-roof displacement loops under different earthquakes with PGA = 0.35 g.

**Fig. 17:**
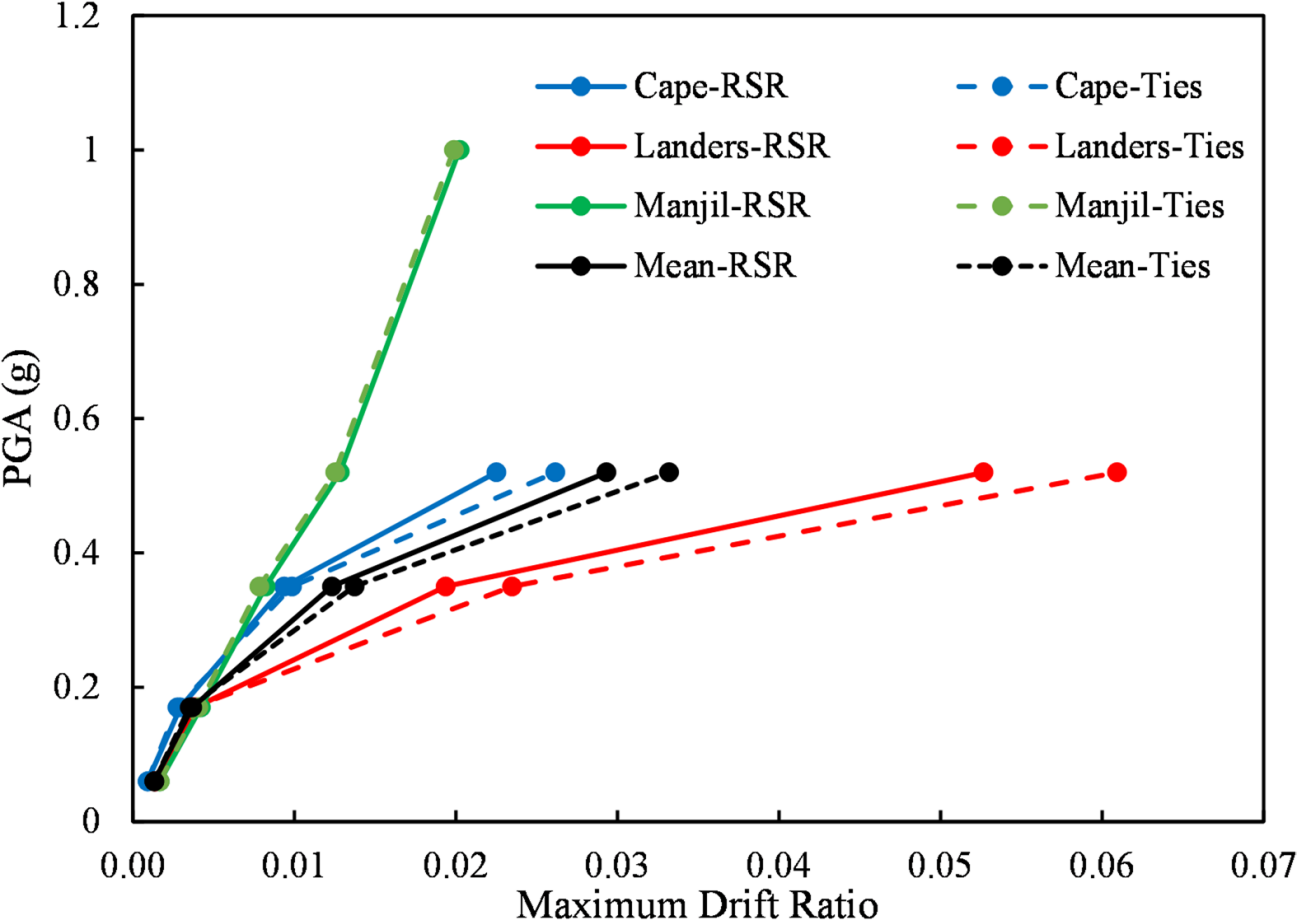
Peak drift of the structure.

Seismic performance results correspond to the model in “Dynamic modeling (seismic loading)”. Following the preliminary validation of the proposed structural model using quasi-static tests (“Quasi-static behavior of RC elements with RSR under lateral loading”, Fig. 15), a series of full-scale dynamic time-history analyses were conducted to evaluate the seismic performance of RC frames confined with RSR compared to conventional transverse reinforcement. The structural model used in this analysis is based on the experimental setup described by Kakaletsis et al.^55^ (see “Quasi-static modeling (lateral loading)” and Fig. 2), consisting of a one-third scale RC frame (1.0 m × 1.5 m), modeled in SAP2000 and scaled to full-size dimensions. The reinforcement layout, concrete strength, boundary conditions, and loading history were replicated based on the specifications of Ref.^55^, ensuring consistency between envelope-based lateral quasi-static and seismic simulations. As an example, Fig. 16 shows the time history displacement curves of the roof of the models under earthquakes with a 475-year return period (PGA = 0.35 g). It can be observed that the frame with RSR confinement shows a relatively slight improvement in terms of displacement values. To further quantify the seismic performance of the RSR-confined frame, base shear–roof displacement loops were shown in Fig. 16. The RSR-confined frame exhibits more stable behavior, characterized by consistent and repeatable loop shapes across successive loading cycles. This stability indicates minimal degradation in strength or stiffness, even under intense seismic demands. These findings underscore the superior stability of quasi-static loops for RSR-confined frames, which is critical for reliable seismic performance under strong ground motions. Table 7; Fig. 17 compare the drift of structural frames with conventional ties and RSR confinement under different earthquake levels. It should be noted that both structures collapsed under the Cape, Mendocino and Landers earthquakes at a 1.00 g scale.

**Table 7:** Comparison of peak roof displacement (m) under different earthquake levels.

PGA (g)	Cape-RSR	Cape-Ties	Landers-RSR	Landers-Ties	Manjil-RSR	Manjil-Ties
0.06	0.002	0.002	0.004	0.004	0.005	0.005
0.17	0.008	0.007	0.011	0.010	0.011	0.011
0.35	0.025	0.027	0.052	0.063	0.022	0.021
0.52	0.061	0.071	0.142	0.165	0.035	0.034
1.00	Failed	Failed	Failed	Failed	0.055	0.054

The average results show a relative improvement due to the RSR confinement, especially at higher earthquake levels. For example, under earthquakes with a peak acceleration of 0.52 g, the use of RSR confinement reduced the displacement response by approximately 8% on average.

### Test results

**Fig. 18:**
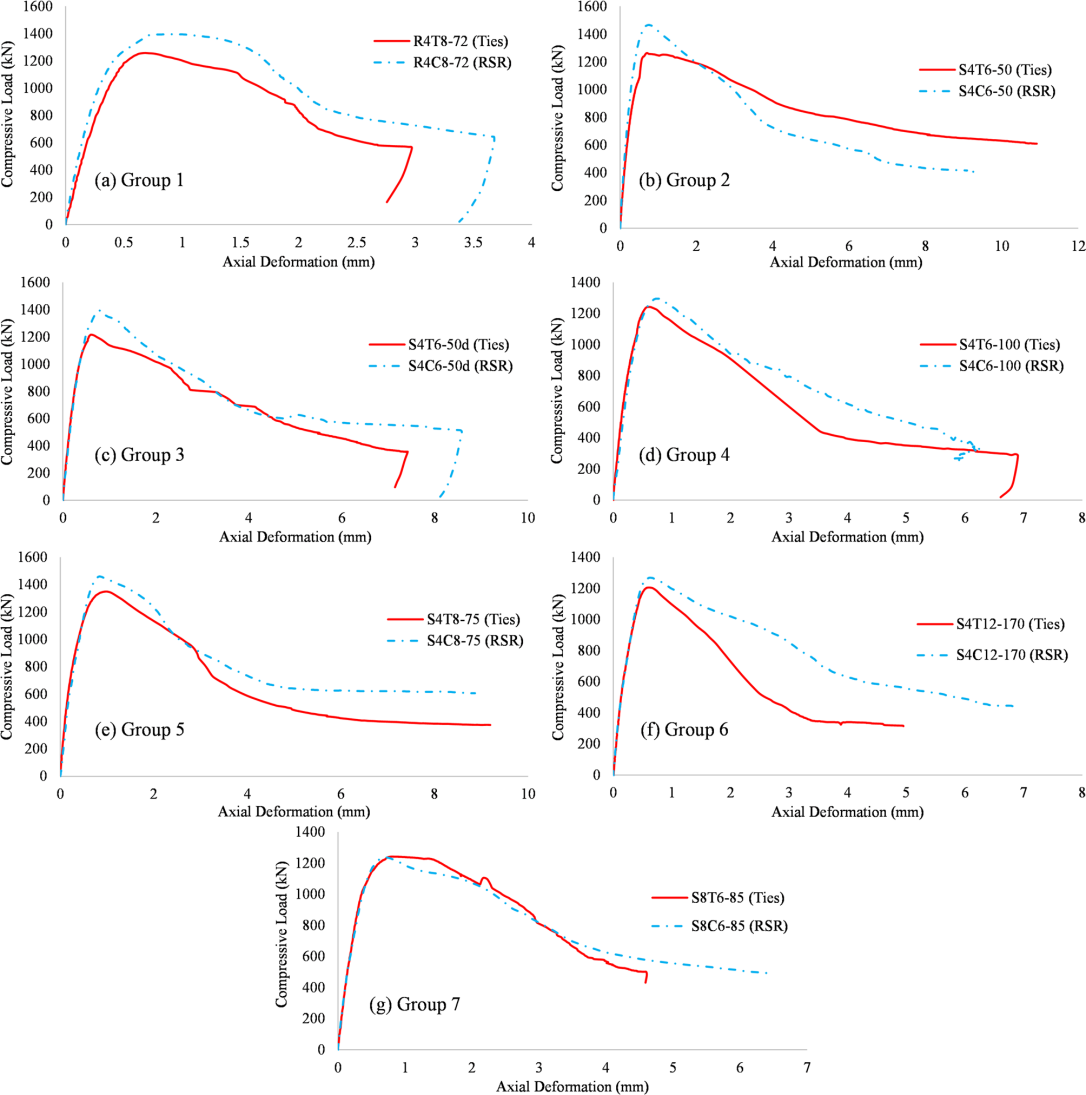
Load-displacement data (displacements are measured over a 260 mm length at the midpoint of the samples).

**Fig. 19:**
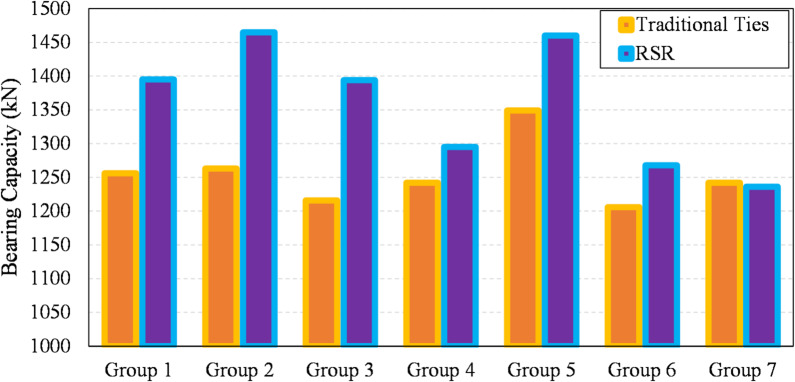
Detailed comparison of the compressive strength across samples in each test group under different earthquake levels.

Experimental findings are reported based on the tests in “Testing program”. The load–deformation outcomes of the specimens under axial compression are presented in Fig. 18a–g, while Fig. 19 provides a comparative strength analysis across groups. Overall, the RSR-confined columns exhibited superior compressive behavior, with an average increase of 8.4% in load-carrying capacity over specimens reinforced with traditional ties. Strength improvements for Groups 1 to 6 were 11.1%, 16.0%, 14.6%, 4.3%, 8.2%, and 5.1%, respectively. These gains can be primarily attributed to the continuous and uniform confinement provided by the RSR configuration, which enhances lateral pressure on the core concrete. The tightly spaced spiral pattern in RSR helps suppress early spalling and delays longitudinal bar buckling, which are critical factors influencing the load-bearing capacity of RC columns. Moreover, the continuity of the RSR helix promotes more efficient stress distribution, particularly in regions away from the loading ends, where conventional ties often allow stress concentration and cracking. The only group that did not follow this trend was Group 7, which showed a marginal strength decrease of 0.5% in the RSR specimen compared to its tie-reinforced counterpart. This anomaly is linked to constructability constraints: the inner reinforcement cage in specimen S8C6-85 had to be slightly reduced in size to accommodate the RSR layout, reducing its confinement efficiency despite identical transverse reinforcement ratio and concrete strength. This geometrical constraint limited the lateral restraint around the concrete core, partially negating the typical benefits of RSR. These findings collectively demonstrate that the RSR system is generally more effective in enhancing compressive capacity—especially when used with adequate spacing and geometry—but its advantages can be offset by practical layout limitations.

**Fig. 20:**
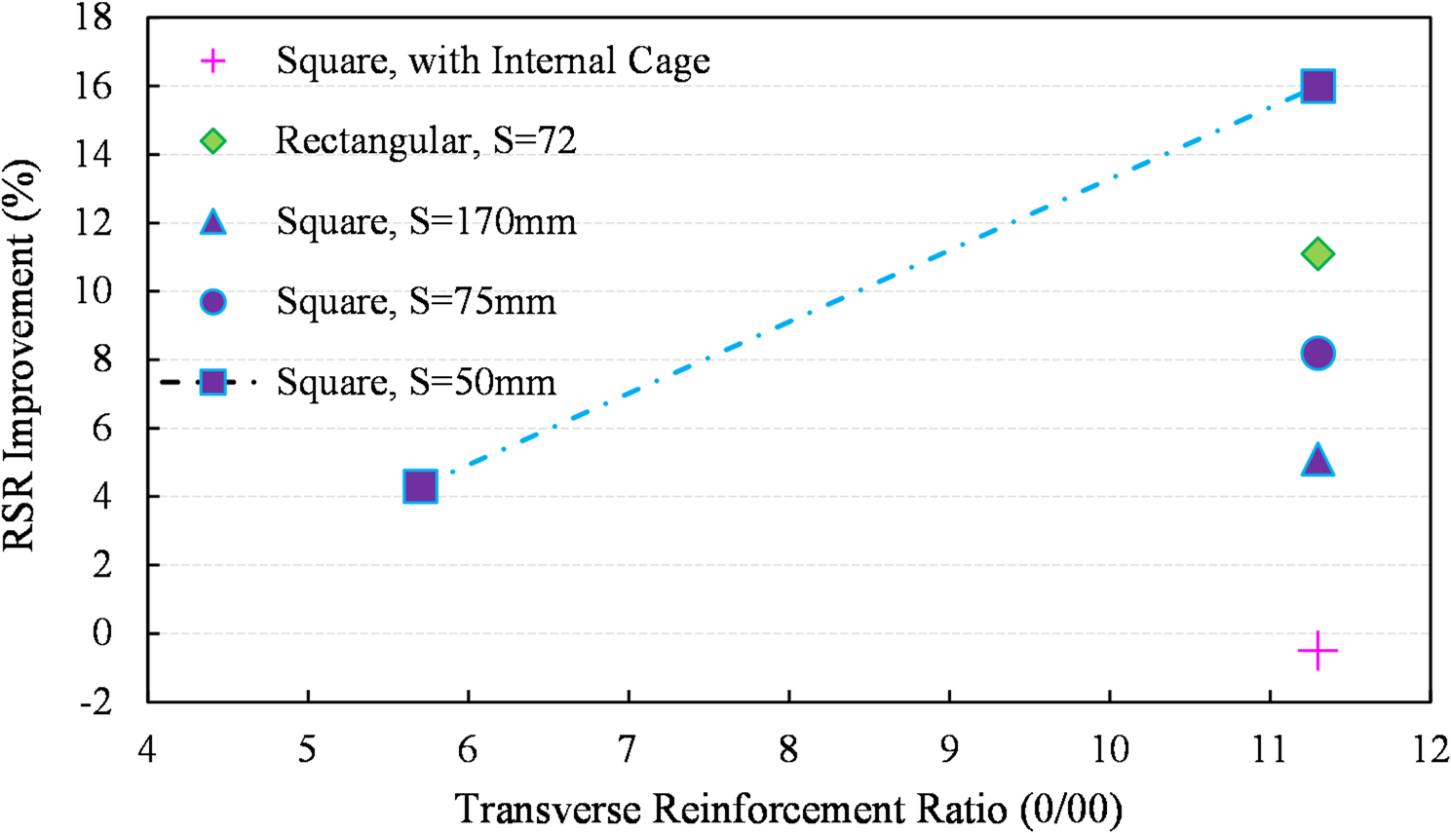
Improvement ratio in capacity for RSR specimens.

Figure 20 illustrates the strength improvement ratios attributed to RSR. The data reveal that the extent of improvement due to RSR is influenced by several factors: the amount of transverse rebar, the pitch of ties (spacing), and the cross-sectional shape. It is evident that increasing the transverse reinforcement ratio and decreasing the spacing significantly enhances this improvement. From the perspective of ductility and post-peak behavior, the findings show no notable disparity between the performance of the two transverse reinforcement configurations. When comparing experimental outputs with non-reduced compressive strength values of *P*_*0*_ and *N* in ACI-CODE-318-19R22 (Table 4), specimens with traditional ties have an average *N/P*_*0*_ ratio of 1.24, while those with RSR have a ratio of 1.35.

Given the inherent continuity of spiral and CTR confinement, it might be suggested that in the event of rupture in transverse rebar, a larger column height would be influenced by stress release in elements with RSR. Consequently, these members might experience a more catastrophic failure compared to those with conventional ties. This hypothesis was tested with specimens from test group 3. A deficiency was intentionally introduced with a single cut in the transverse reinforcement at a specific location, as shown in Fig. 5e. Figure 18c demonstrates that this prediction did not materialize. While RSR resulted in a significant strength increase, the load-deformation curves exhibited negligible disparity in their descending branches.

**Fig. 21:**
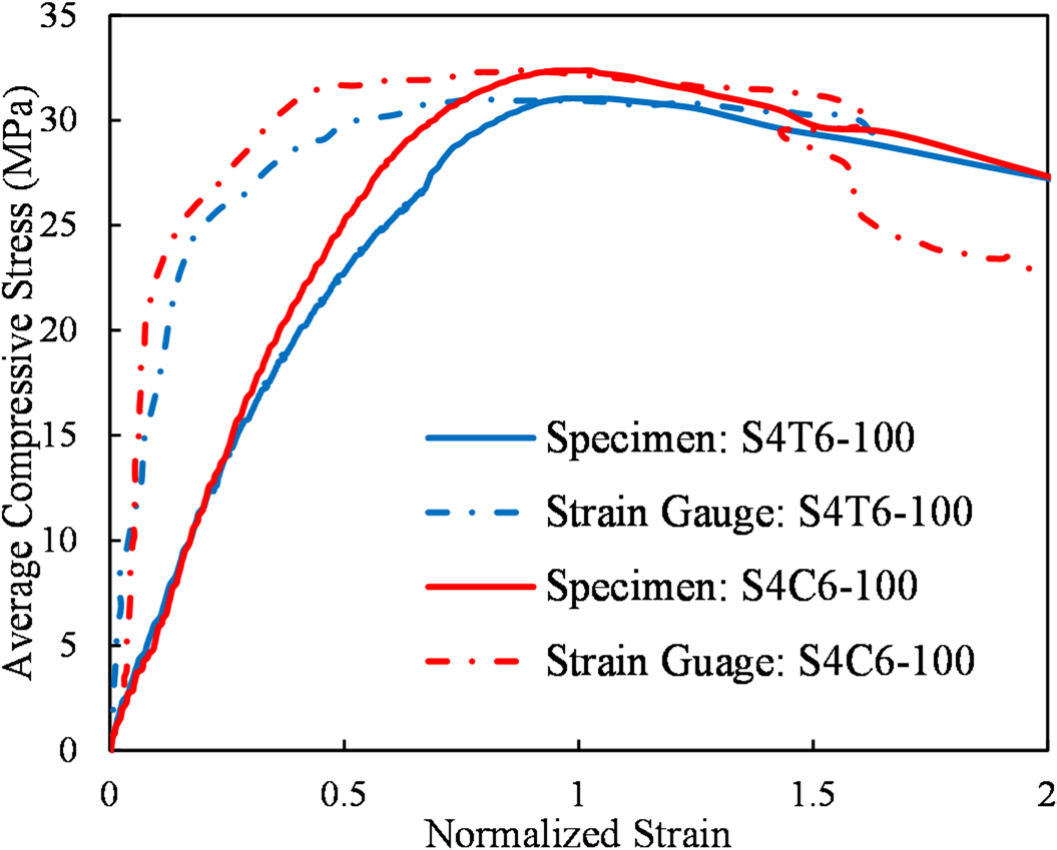
Comparison of average compressive stress versus normalized strain for specimens S4T6-100 and S4C6-100.

Figure 21 displays the compressive stress vs. normalized strain results for both the specimens and the strain gauges attached to the transverse reinforcement. Compressive stress applied to the samples is shown on the vertical axis, while normalized strain is on the horizontal axis (*ε*_*sg*_*/ε*_*yh*_ for strain gauges, where *ε*_*yh*_ is the yield strain for transverse rebar, *ε*_*sg*_ is the strain determined by the gauge, and *ε*_*yh*_*/ε*_*cc*_ for specimens, where *ε* is the sample’s compressive strain and *ε*_*cc*_ is the peak compressive strain). The strain gauges were installed at the mid-height of the columns on the external surface of the ties (for S4T6-100) and RSR (for S4C6-100). The solid lines correspond to the stress–strain response of the concrete specimens, while the dashed lines show the strain gauge measurements on the confining reinforcement. This figure helps illustrate how lateral confinement behavior correlates with axial performance in different reinforcement configurations.

The data presented in Fig. 21 suggests:

- During the initial loading stages, the lateral expansion of the concrete specimens induces a measurable strain in the transverse reinforcement.
- A critical point exists, termed the “concrete dilatation brink” at which the transverse reinforcement becomes entirely engaged. With further load increments, the strain ratio in the reinforcement experiences a significant increase.
- The peak load is attained prior to the yielding of the transverse reinforcement in either test specimen. It is important to acknowledge that the placement of the strain gauges may not have necessarily coincided with the critical stress location within the rebars.
- For a given load level, RSR generally exhibits a modestly lower strain ratio compared to traditional ties (e.g., 0.14 for RSR vs. 0.19 for ties at 25 MPa). This observation, coupled with the well-established benefit of RSR in providing a more uniform confinement stress distribution throughout the concrete core (a fundamental assumption of the proposed theoretical method), suggests that RSR offers superior load-carrying capacity.

**Fig. 22:**
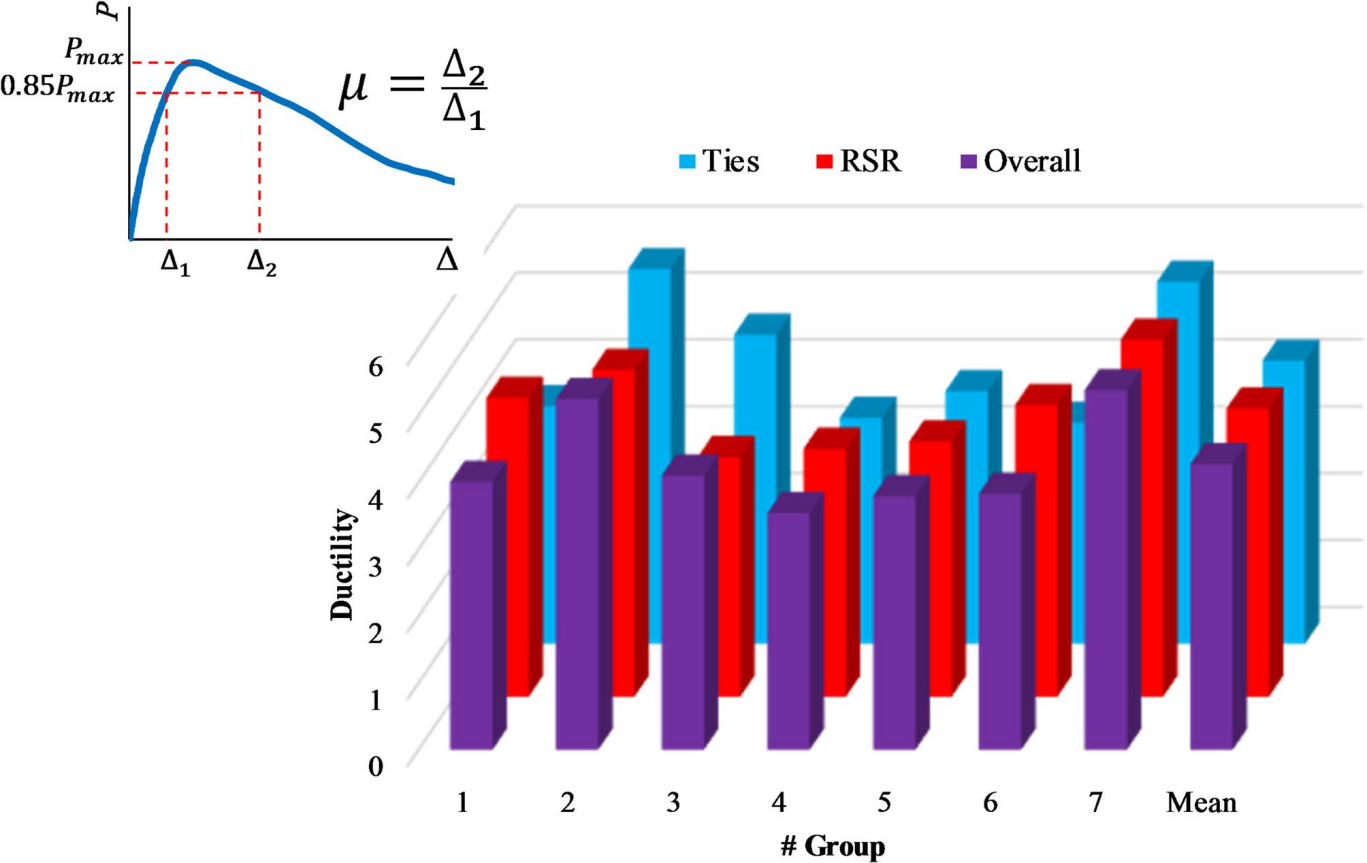
The ductility values of the specimens for different test groups.

One significant aspect of the samples’ behavior that affects the seismic performance of the structure is their ductility. Figure 22; Table 8 present the ductility values of the samples in each test group. In our analysis, ductility (µ) is calculated as the ratio of ultimate displacement (Δ_2_​) to yield displacement (Δ_1_​), as shown in Fig. 22. This approach aligns with conventional structural engineering practices for assessing deformation capacity (e.g., as described in [62,63]). A careful examination of these results reveals that, in terms of ductility, there is no substantial difference between samples with rectangular ties and those with RSRs. On average, samples with RSR have about 2% more ductility. It is important to note that the amount of transverse reinforcement used in the samples with RSR is, on average, about 12% less than that in similar samples with conventional transverse tie reinforcement. This relatively similar performance, combined with reduced reinforcement usage, highlights the relative advantage of the RSR. The experimental results strongly indicate that RSR’s continuous nature and inclination angle demonstrably enhance the uniformity of confining stress distribution within the concrete core. This results in lower strain ratios under certain compressive loads compared to conventional ties, thereby significantly enhancing the load-carrying capacity of the confined concrete. However, in the descending branch, RSR confinement tends to cause asymmetric failure modes under large axial deformations. While its efficacy relative to alternative confinement strategies remains inconclusive, it warrants further investigation to elucidate its potential benefits and drawbacks comprehensively.

Moreover, the anticipated catastrophic failure mode for RSR-confined columns in the event of transverse reinforcement rupture was not observed. Given the numerous advantages of RSR—such as accelerated and simplified reinforcement construction, reduced rebar usage, decreased likelihood of construction errors, and overall strength improvement—for RC columns with rectangular cross-sections the adoption of CTR is highly suggested. While the existing design methods appear sufficient for these members, the application of increased strength reduction factors (permissible for circular spirals) is discouraged.

**Table 8:** Comparison of the ductility values for the samples.

Group #	Ties	RSR	Overall	RSR/ties
1	3.55	4.47	4.01	1.26
2	5.61	4.89	5.25	0.87
3	4.62	3.59	4.11	0.78
4	3.38	3.71	3.54	1.10
5	3.78	3.82	3.80	1.01
6	3.30	4.36	3.83	1.32
7	5.42	5.34	5.38	0.99
Mean	4.24	4.31	4.27	1.02
Covariance	0.21	0.14	0.16	0.18

### Analytical results

**Fig. 23:**
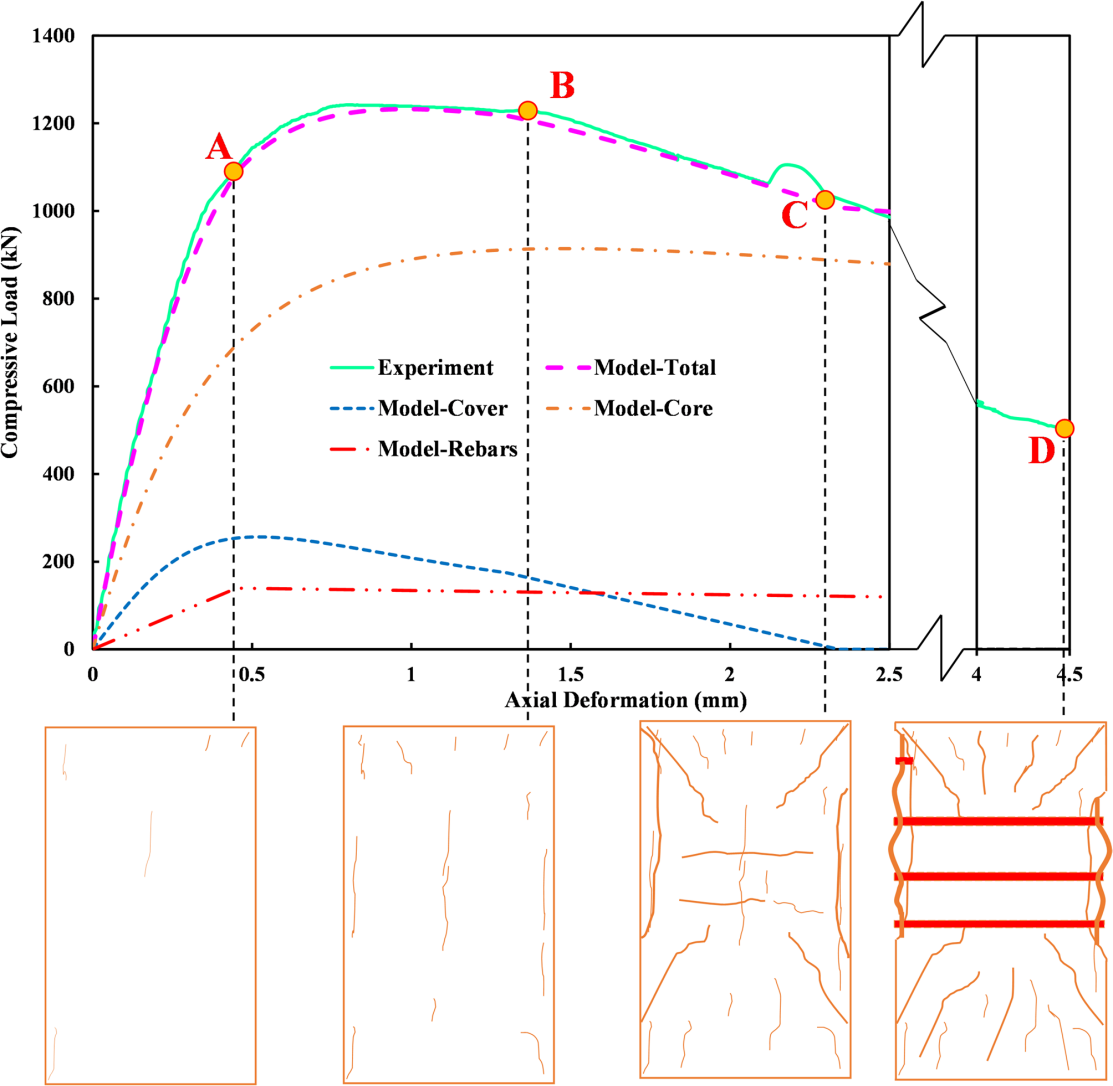
Impact of each component on the compressive performance for sample S8T6-85, highlighting damages at critical loading steps.

**Fig. 24:**
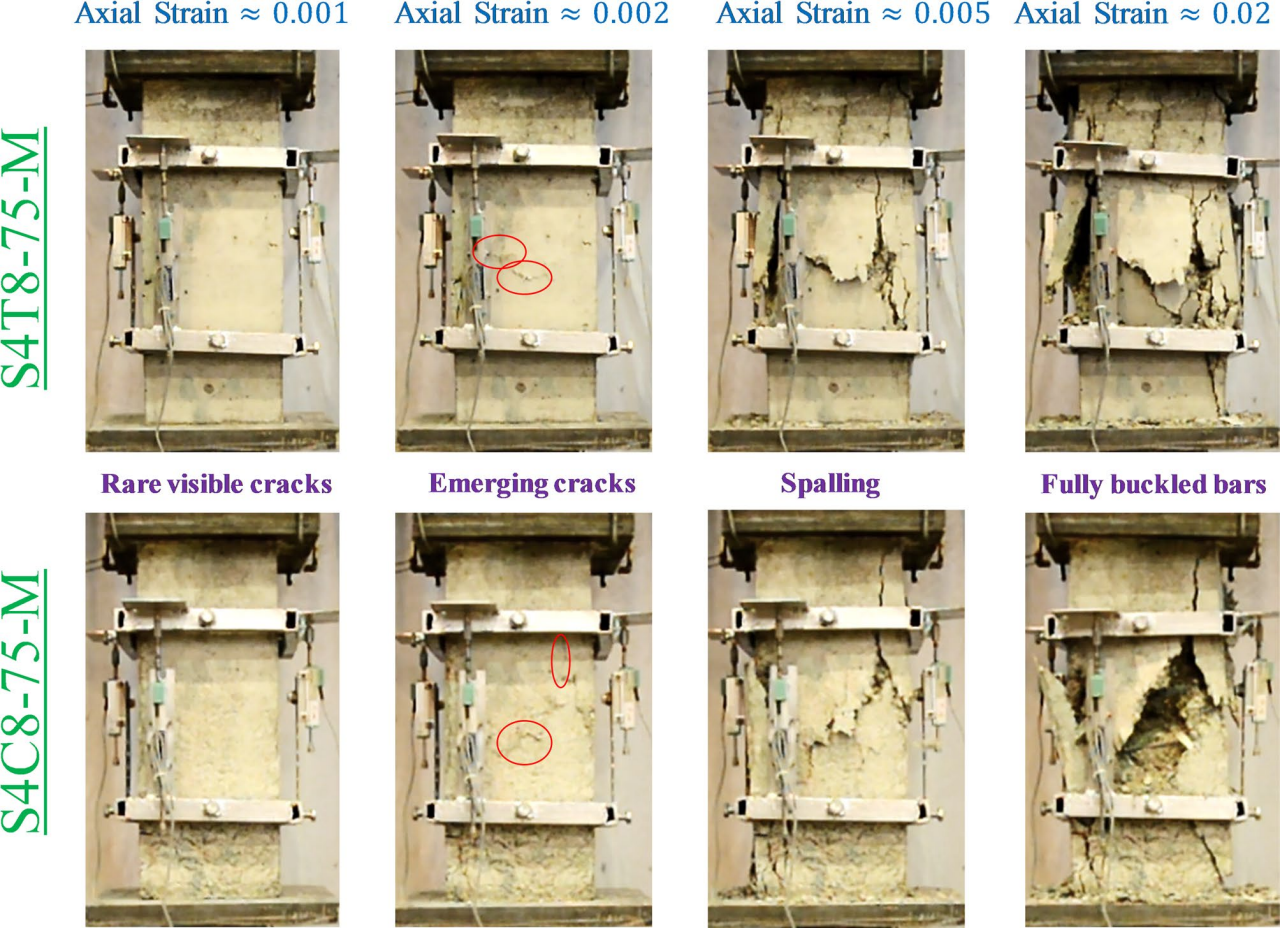
The progression of damage in the samples.

**Fig. 25:**
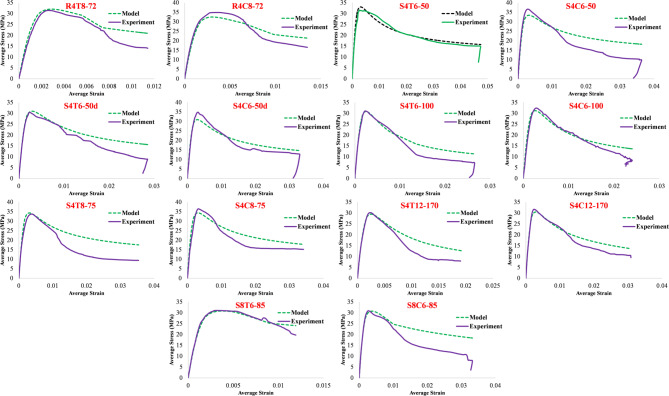
Comparison of proposed analytical model and experimental results.

Analytical predictions are derived from the model in “Mander et al. [49] model and proposed analytical model”. Using the aforementioned approaches for each component, the compression performance of the tested columns can be accurately determined using Eqs. (2)–(6) and Eqs. (A1)–(A11). Figure 23 presents a comparison between the behavior of specimen S8T6-85 as predicted by the proposed method and as observed in the experiment. The illustration elucidates the individual component contributions to the material’s overall compressive response. Additionally, it depicts the crack patterns and progressive evolution of damage under varying loading conditions. At Node A, the Dhakal model [61] indicates that the longitudinal reinforcements yield, with sparse visible cracks in the sample. At Node B, cover concrete cracking increases significantly, ending at Node C where spalling is complete. Node D represents the final experimental stage, characterized by complete deterioration of the mid-zone cover concrete and prominent buckling of the longitudinal rebars. The damage development trend for the samples is largely similar to each other. For example, Fig. 24 shows this trend for samples S4T8-75 and S4C8-75-M. As observed, around an axial average strain of 0.002, cracks are clearly visible. The rate of increase and development of cracks intensifies rapidly after this point, and concrete spalling becomes apparent. By a strain of about 0.005, the concrete cover has practically separated and lost its contribution to the member’s behavior. From this point onward, the effect of rebar buckling gradually becomes apparent, and at higher strains, around 0.02, this buckling can be observed in its severe form. Ultimately, Fig. 25 compares the test outcomes with those determined from the proposed model for all specimens. The comparison demonstrates a remarkable agreement, validating the accuracy of the proposed method. To quantitatively evaluate the accuracy of the proposed method for calculating the compressive capacity of members, the capacity ratio values (the ratio of experimental capacity to theoretical capacity) of reinforced concrete compressive samples were presented in Table 9. The average capacity ratio for samples with RSR is 1.03, while for samples with conventional ties, it is 0.99. Given that the overall average and standard deviation for all samples are 1.01 and 1%, respectively, it can be stated that the proposed method has acceptable accuracy in predicting the capacity of the samples.

As explained in “Mander et al. [49] model and proposed analytical model”, the Mander model accounts for arching effects both laterally and vertically. The fundamental difference between the behaviors of RSR and Ties lies in these arching effects, which are addressed in Eqs. (A7) and (2). In commercial software such as SAP2000, where it is not possible to modify behavioral models, a simple approximation of the problem can be achieved by adjusting the amount of transverse reinforcement in the confined concrete core using the coefficient mentioned in the Eq. (1). The rationale behind this adjustment is understandable based on the modified Mander equations presented in the mentioned section.

The novelty of Eq. (1) lies in its application to continuous RSR—a configuration not covered by existing design guidelines or classical models like Mander’s. Unlike traditional tie reinforcement, the RSR system introduces continuous three-dimensional confinement, making bond-slip and stress redistribution smoother. Our proposed η_RCTR_ accounts for this behavior in a simplified form, making it practical for implementation in structural modeling platforms like SAP2000. This approach enables meaningful simulation of global frame behavior under seismic loading, which we validated against experimental results (see Fig. 15).

**Table 9:** Comparison of experimental to theoretical compressive capacity ratio.

Group #	Ties	RSR	Overall
1	0.98	1.04	1.01
2	0.95	1.06	1.01
3	0.98	1.08	1.03
4	1.01	1.02	1.01
5	0.98	1.04	1.01
6	1.02	1.02	1.02
7	1.01	0.97	0.99
Mean	0.99	1.03	1.01
Standard deviation	2.1	3.4	1.3

## Conclusions

This study investigated the static and dynamic behavior of RC elements confined with RSR, a form of continuous CTR, through experimental tests, numerical simulations, and theoretical modeling. The key findings are summarized as follows:

- Compressive performance: RSR-confined specimens exhibited an average of 8.4% higher load-carrying capacity than those with conventional ties, with more uniform stress distribution and delayed spalling and bar buckling. Numerical analyses using the Concrete Damaged Plasticity model also confirmed these improvements in confinement and strength retention.
- Shear behavior: Computational models and standards (SMCFT, MPLANE, VecTor2, CEB-FIP, CSA, AASHTO) overestimated the shear strength. No significant difference in shear cracking angle was observed between ties and RSR, but RSR exhibited blunter crack propagation. SMCFT underestimated transverse reinforcement’s shear capacity enhancement, while MPLANE/VecTor2 improved predictions.
- Seismic and envelope-based lateral quasi-static response: Under high-intensity earthquakes (PGA = 0.52 g), frames with RSR confinement showed an 8% average reduction in displacement, indicating enhanced seismic resilience.
- Ductility and efficiency: While ductility enhancement was modest (~ 2%), the volume of transverse reinforcement in RSR specimens was about 12% lower, highlighting material efficiency.
- Analytical modeling: An extended version of Mander’s confined concrete model was developed and successfully applied to RSR-confined columns, yielding close agreement with test results (average prediction ratio = 1.03).

This study assessed lateral static performance based on envelope curves obtained from monotonic analyses. No cyclic simulations were performed. While this approach captured essential performance metrics such as strength and drift capacity, it did not fully represent pinching effects. Nevertheless, the results confirm that RSR provides a promising alternative to traditional ties, enhancing strength and constructability without compromising seismic or post-peak performance.

## Supplementary Information

Below is the link to the electronic supplementary material.

Supplementary Material 1

## Data Availability

All data generated or analyzed during this study are included in this published article.
