# Supplementary material for: Numerical experimental and theoretical investigation of reinforced concrete elements with rectangular spiral rebar for multi-behavior analysis
Source: Sci Rep. 2025 Nov 22;15:45015. doi: 10.1038/s41598-025-29097-w (PMC12749287; doi:10.1038/s41598-025-29097-w)
Supplement: Supplementary file 1 — Supplementary Material 1 [file 41598_2025_29097_MOESM1_ESM.pdf]

Appendix A: Mander model formulations

This model stands out for its exceptional precision, simplicity, and robust physical basis, earning widespread recognition among researchers and engineers. Despite its simplicity, Mander confined concrete model follows a physics-based approach in its solution process, making it a suitable model for better understanding the differences in performance between tied reinforcement and closed stirrups. Mander et al.'s model considers the difference between ties and stirrups by focusing on the arching effect in the effective core area of the concrete. In this model, for the CTR system, a larger portion of the concrete core is classified as the confined stress region. The critical relationships of Mander's model are encapsulated in Eqs. (A1)-(A11) and Table A1.

$$f_c = \frac{f'_{cc} x r}{r - 1 + x^r} \tag{A1}$$

$$x = \frac{\varepsilon_c}{\varepsilon_{cc}} \tag{A2}$$

$$\varepsilon_{cc} = \varepsilon_{co}\left[1 + 5\left(\frac{f'_{cc}}{f'_{co}}\right) - 1\right] \tag{A3}$$

$$r = \frac{E_c}{E_c - E_{\sec}} \tag{A4}$$

$$E_c = 5000\sqrt{f'_{co}} \tag{A5}$$

$$E_{\sec} = \frac{f'_{cc}}{\varepsilon_{cc}} \tag{A6}$$

$$k_e = \frac{\left(1 - \sum \frac{\left(w'_i\right)^2}{6 b_c d_c}\right)\left(1 - \frac{s'}{2 b_c}\right)\left(1 - \frac{s'}{2 d_c}\right)}{\left(1 - \rho_{cc}\right)} \tag{A7}$$

$$s' = s - d_{tr} \tag{A8}$$

$$\rho_x = \frac{A_{sx}}{s d_c}, \rho_y = \frac{A_{sy}}{s b_c} \tag{A9}$$

$$f'_{lx} = k_e \rho_x f_{yh}, f'_{ly} = k_e \rho_y f_{yh} \tag{A10}$$

$$f'_{cc} = f'_{co}\left(-1.254 + 2.254\sqrt{1 + \frac{7.94 f'_l}{f'_{co}}} - 2\frac{f'_l}{f'_{co}}\right) \tag{A11}$$

Table A1. Mander model formulation

Symbol	Parameter descriptions
f_{cc}, f_{co}	Compressive strength of unconfined and confined concrete, respectively
ε_{cc}, ε_{co}	Longitudinal compressive strain corresponding to above stresses
E_c	Tangent modulus of elasticity of concrete
k_e	Confinement Effectiveness coefficient
w_i	Net spacing of effectively braced longitudinal rebars
S, d_{tr}	Spacing and diameter of transverse reinforcement, respectively
b_c, d_c	Core dimensions
A_{sx}, A_{sy}	Total area of transverse bars running in the x and y directions, respectively
ρ_{cc}	The ratio of longitudinal reinforcement to the area of core section
f_l	Lateral confining pressure on concrete
f_{yh}	Yield strength of transverse reinforcement
